# Distinct immune landscapes characterize highly versus minimally invasive brain metastases

**DOI:** 10.1172/jci.insight.199498

**Published:** 2026-05-22

**Authors:** Sarah M. Maritan, Elham Karimi, Matthew Dankner, Aldo Hernandez-Corchado, Miranda W. Yu, Matthew G. Annis, Yashar Aghazadeh Habashi, Morteza Rezanejad, Bridget Liu, Nebras Koudieh, Emilie Pichette, Parvaneh Fallah, Benoit Fiset, Yuhong Wei, Ali Nehme, Chun Geun Lee, Jack A. Elias, Morag Park, Yasser Riazalhosseini, Hamed Najafabadi, Kevin Petrecca, Marie-Christine Guiot, Daniela F. Quail, Logan A. Walsh, Peter M. Siegel

**Affiliations:** 1 Goodman Cancer Institute,; 2 Department of Medicine, Division of Experimental Medicine,; 3 Department of Human Genetics, Faculty of Medicine and Health Sciences,; 4 Victor Phillip Dahdaleh Institute of Genomic Medicine, and; 5 Department of Physiology, Faculty of Medicine and Health Sciences, McGill University, Montreal, Quebec, Canada.; 6 Research Institute of McGill University Health Centre, Montreal, Quebec, Canada.; 7 Department of Pathology, Faculty of Medicine and Health Sciences, and; 8 Gerald Bronfman Department of Oncology, McGill University, Montreal, Quebec, Canada.; 9 Segal Cancer Centre, Jewish General Hospital, Montreal, Quebec, Canada.; 10 Intercollege, Hanyang University, Seoul, Korea.; 11 Division of Medicine and Biological Sciences, Brown University, Providence, Rhode Island, USA.; 12 Department of Oncology, Faculty of Medicine and Health Sciences, McGill University, Montreal, Quebec, Canada.; 13 Montreal Neurological Institute-Hospital, McGill University Health Centre, Montreal, Quebec, Canada.; 14 Department of Neurology and Neurosurgery, Faculty of Medicine and Health Sciences, McGill University, Montreal, Quebec, Canada.

**Keywords:** Immunology, Oncology, Brain cancer

## Abstract

Brain metastases (BrMs) occur in approximately 30% of cancer patients, causing nearly one-fifth of cancer deaths. While immune checkpoint inhibitors (ICIs) benefit some BrM patients, responses remain highly variable. This variability partly reflects distinct histopathological growth patterns that include minimally invasive (MI) and highly invasive (HI) brain BrMs. Here we show that MI BrMs exhibit robust immune infiltration, whereas HI lesions are immunosuppressed. However, histological differentiation between MI and HI can be challenging because of subjective margin assessment. Here, using highly multiplexed spatial proteomics on 119 tumor sections from 46 patients with BrMs, we identify CHI3L1 as a key mediator of the immunosuppressive microenvironment in HI BrMs. In preclinical models, genetic deletion of CHI3L1 converts immune-cold metastases into lymphocyte-rich, ICI-responsive lesions infiltrated by granzyme B^+^ CD8^+^ T cells. In BrM patients treated with ICI, immunohistochemical quantification of CHI3L1 expression was a stronger predictor of ICI response than traditional MI/HI classification. Thus, CHI3L1 represents a promising biomarker and therapeutic target for BrMs.

## Abstract 2

Invasive brain metastases with high astrocyte-derived CHI3L1 are immunosuppressed. Loss of CHI3L1 restores T cell infiltration, which improves response to immunotherapy

## Graphical abstract

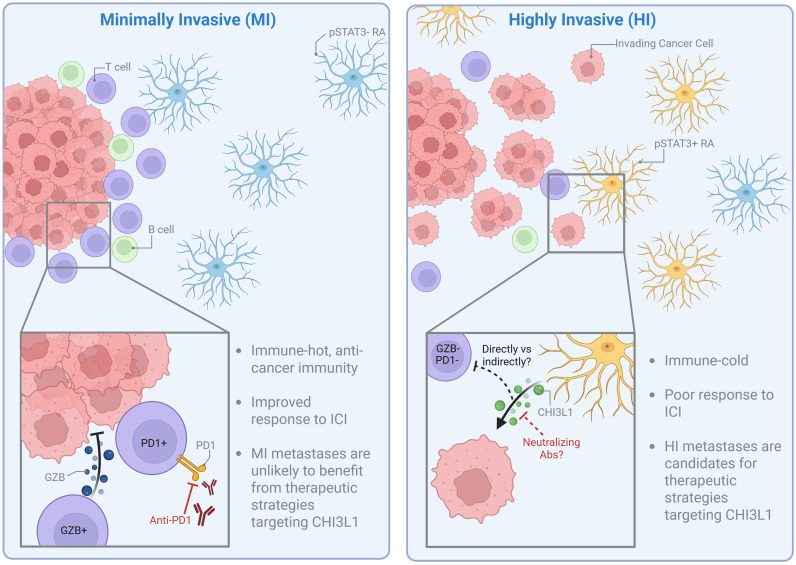

## Introduction

Cancer metastasis to the brain is a complication of advanced cancer that is increasing in incidence (1, 2). Brain metastases (BrMs) are estimated to affect approximately 30% of patients with advanced solid tumors, predominantly arising from tumors of the breast, lung, and skin (melanoma). Although prognosis following a BrM diagnosis continues to be generally poor, there are various clinical and biological factors that have been associated with improved patient outcomes. These include the presence of targetable alterations (2, 3), well-controlled extracranial disease (1, 3), and the absence of cancer cell invasion into the peritumoral brain parenchyma (4, 5). Indeed, highly invasive (HI) growth of BrMs has been described by several groups (4–8) as being associated with local recurrence and decreased overall survival compared with well-circumscribed, minimally invasive (MI) lesions (4, 5).

Recent insights into the microenvironment of brain tumors, including BrMs, have revealed the presence and anticancer functions of immune cells, breaking the previously held dogma of the brain being an immune-isolated organ (9). Indeed, recent studies reveal that breast cancer BrMs downregulate MHC class I expression and components of the antigen-processing machinery, mediated in part by metastasis-associated astrocytes (10, 11). Such studies have supported the use of immunotherapeutic agents in treating BrMs, particularly immune checkpoint inhibitors (ICIs), which are now widely used in patients with BrMs from melanoma and non–small cell lung cancer (NSCLC) (1, 3). However clinical studies continue to reveal highly variable results, with ICIs benefiting only a subset of patients with BrMs. For example, phase II trials of single-agent cytotoxic T lymphocyte antigen-4 (CTLA-4) or programmed cell death protein-1 (PD-1) inhibitors in patients with melanoma BrMs revealed intracranial response rates of only 10%–24% (12, 13). Similarly, PD-1 inhibition in patients with brain-metastatic NSCLC or diverse histopathologies revealed intracranial response rates of 33% and 9%, respectively (13, 14). Although combinatorial CTLA-4 and PD-1 inhibition provided intracranial response in up to 55% of patients with melanoma BrMs (15, 16), there continues to be limited understanding of the underlying biology that determines why only a subset of patients respond. Thus, there is growing interest in further evaluating the tumor immune microenvironment of BrMs to elucidate biomarkers or mechanisms that contribute to ICI resistance.

Spatial profiling is emerging as a powerful approach to characterize the microenvironments of BrMs (17–19). Here, we build on our previous work that profiled the spatial immune landscapes of primary brain tumors and BrMs using imaging mass cytometry (IMC) (20), with a specific focus on MI and HI lesions. We reveal that MI BrMs are characterized by lymphoid-rich multicellular neighborhoods at the brain-tumor interface, which are largely absent in HI BrMs. Our findings implicate chitinase 3–like-1 (CHI3L1) as a key factor involved in orchestrating the immune-cold environment of HI BrMs and identify CHI3L1 as a biomarker of patients who are unlikely to respond to current ICI therapies. Together, this work highlights the diverse immunological architecture of BrM subtypes, which can be stratified by the degree of parenchymal invasion exhibited by these lesions. These results suggest that the immune system may modulate the invasive growth of BrMs and that CHI3L1 may serve as a clinically relevant therapeutic target to improve response to ICI.

## Results

### IMC reveals distinct immunological landscapes between MI and HI BrMs.

**Figure 1:**
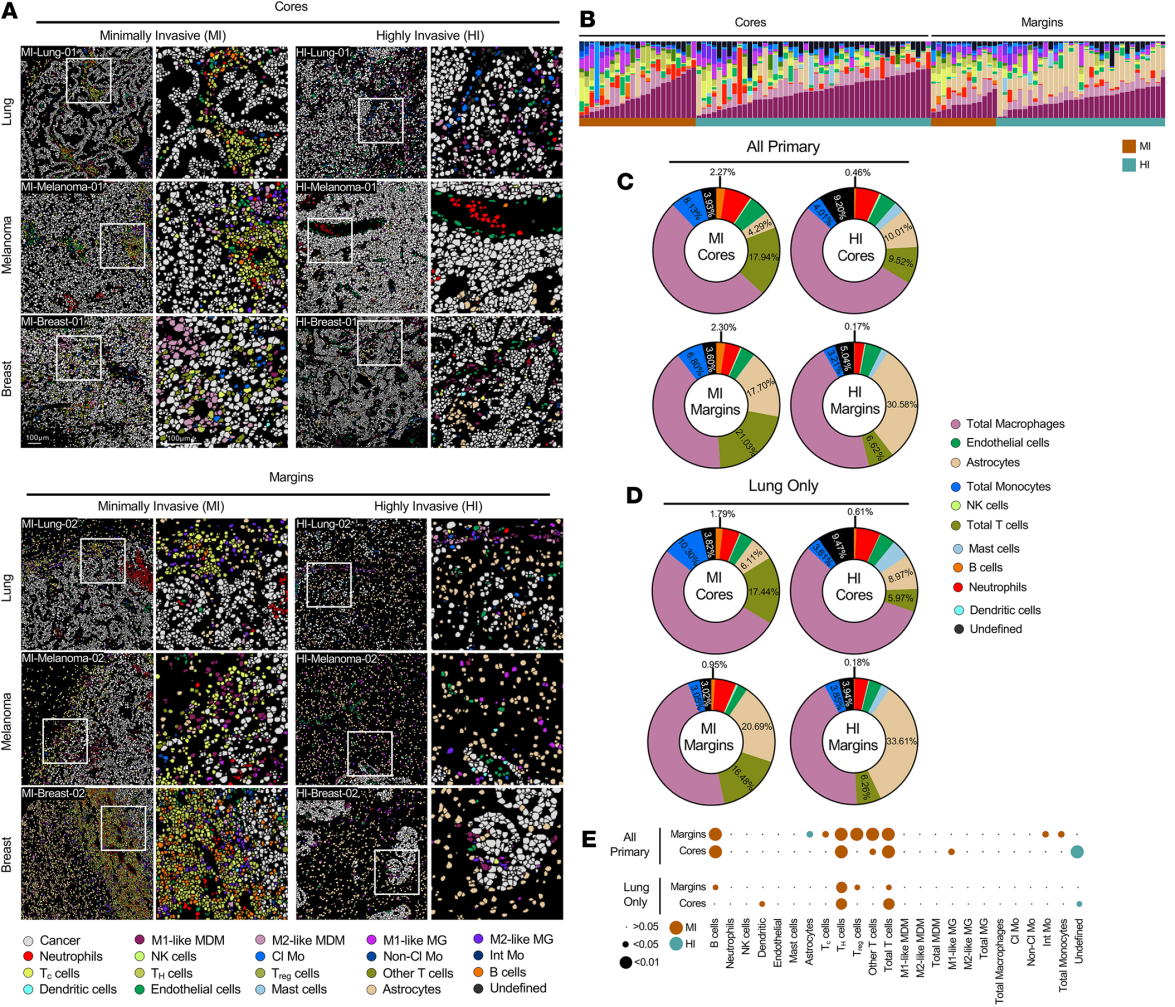
MI BrMs are characterized by a greater degree of immune infiltration when compared with HI lesions. (**A**) Representative images illustrating cell lineage assignments from imaging mass cytometry (IMC) data are shown for the center (Cores) and brain-tumor interface (Margins) of patient BrMs. Examples from lung cancer (top), melanoma (middle), and breast cancer (bottom) are provided, and representative images of MI and HI BrMs are shown. High-magnification images, denoted by white squares, are provided to the right of each low-magnification image. The color code for lineage assignment is provided below. Scale bars: 100 μm (all images). (**B**) Distribution of cell populations as a percentage of stroma in the tumor microenvironment, sorted by sampling region (cores, left; margins, right) and invasion pattern (MI, brown; HI, blue). Cell frequencies in each image are displayed by the vertical bars; color code corresponds to cell lineages described in **A**. (**C** and **D**) Cell populations as a percentage of stroma in the tumor microenvironment for cores and margins of HI and MI BrMs from all primary sites (**C**) or only lung cancer (**D**). Color codes for each cell assignment are shown below. (**E**) Bubble plot depicting cell frequency as a proportion of stromal content between HI and MI BrMs from all primary sites (top) or only lung cancer (bottom). Both core and margin samples are shown. Bubble size indicates *P* value; bubble color indicates the invasion pattern that exhibits the greater representation of the indicated cell population. Data (**C**–**E**) correspond to Supplemental Figure 1, B and C. Cl Mo, classical monocyte; non-Cl Mo, nonclassical monocyte; Int Mo, intermediate monocyte.

To comprehensively profile the cellular composition and spatial organization of BrMs, we leveraged our previously published cohort of BrM samples that underwent IMC (20). This cohort includes 119 histopathological images from 46 patients with BrMs from various primary sites (*n* = 51 lung, *n* = 29 breast, *n* = 19 melanoma, *n* = 20 other). Patient-matched images of the center of the metastatic lesion (core) and the brain-tumor interface (margin) were included when possible (20). Highly multiplexed staining and subsequent cell segmentation identified 20 unique cell types representing a variety of lymphoid, myeloid, and structural (e.g., endothelial, astrocytic, cancer) cell populations (Figure 1, A and B) (20).

Following classification by histopathological growth pattern (Supplemental Figure 1A; supplemental material available online with this article; https://doi.org/10.1172/jci.insight.199498DS1) (4), and amalgamating BrMs from all primary sites, we observed elevated frequencies of B and T cells in MI BrM cores and margins, and undefined cells in HI BrM cores (Figure 1C and Supplemental Figure 1B). To avoid potential confounding issues associated with different primary cancer types that have spread to the brain, we sought to perform a focused analysis on BrMs originating from a specific primary tumor type. While melanoma and breast cancer BrMs lacked sufficient samples to perform primary tumor–specific analyses, the lung cancer BrM cohort possessed samples that were more evenly distributed between MI and HI lesions (MI: *n* = 9 cores, *n* = 6 margins; HI: *n* = 20 cores, *n* = 16 margins), thus enabling further analysis (Supplemental Figure 1A). Across lung cancer BrM cores, MI lesions possessed higher frequencies of dendritic cells, T helper (T_H_) cells, and total T cell populations compared with HI samples, which were enriched in cells that were undefined by our antibody panel (“undefined”) (Figure 1D and Supplemental Figure 1C). When the lung cancer BrM margins were evaluated, B cells, T_H_ cells, regulatory T (T_reg_) cells, and total T cells were more abundant in MI versus HI samples (Figure 1D and Supplemental Figure 1C). These data argue that our findings are representative of the MI growth pattern independent of primary source (Figure 1E). We additionally observed that astrocytes were more abundant in HI versus MI margins, consistent with previous work implicating astrocytes in cancer cell invasion in the brain (Figure 1, C–E) (8, 21–23). Intriguingly, T_reg_ and cytotoxic T (T_C_) cells were found to be enriched in MI versus HI BrMs only at the tumor margin, while T_H_ cells and Other T cells (CD3^+^, CD4^–^, and CD8^–^) were enriched in both the cores and margins of MI versus HI samples (Figure 1, C–E). These data demonstrate enrichment of lymphocyte populations in MI compared with HI BrMs and are suggestive of an immune-hot microenvironment in MI lesions.

### Spatial transcriptomics analysis confirms that an inflammatory microenvironment is associated with MI BrMs.

**Figure 2:**
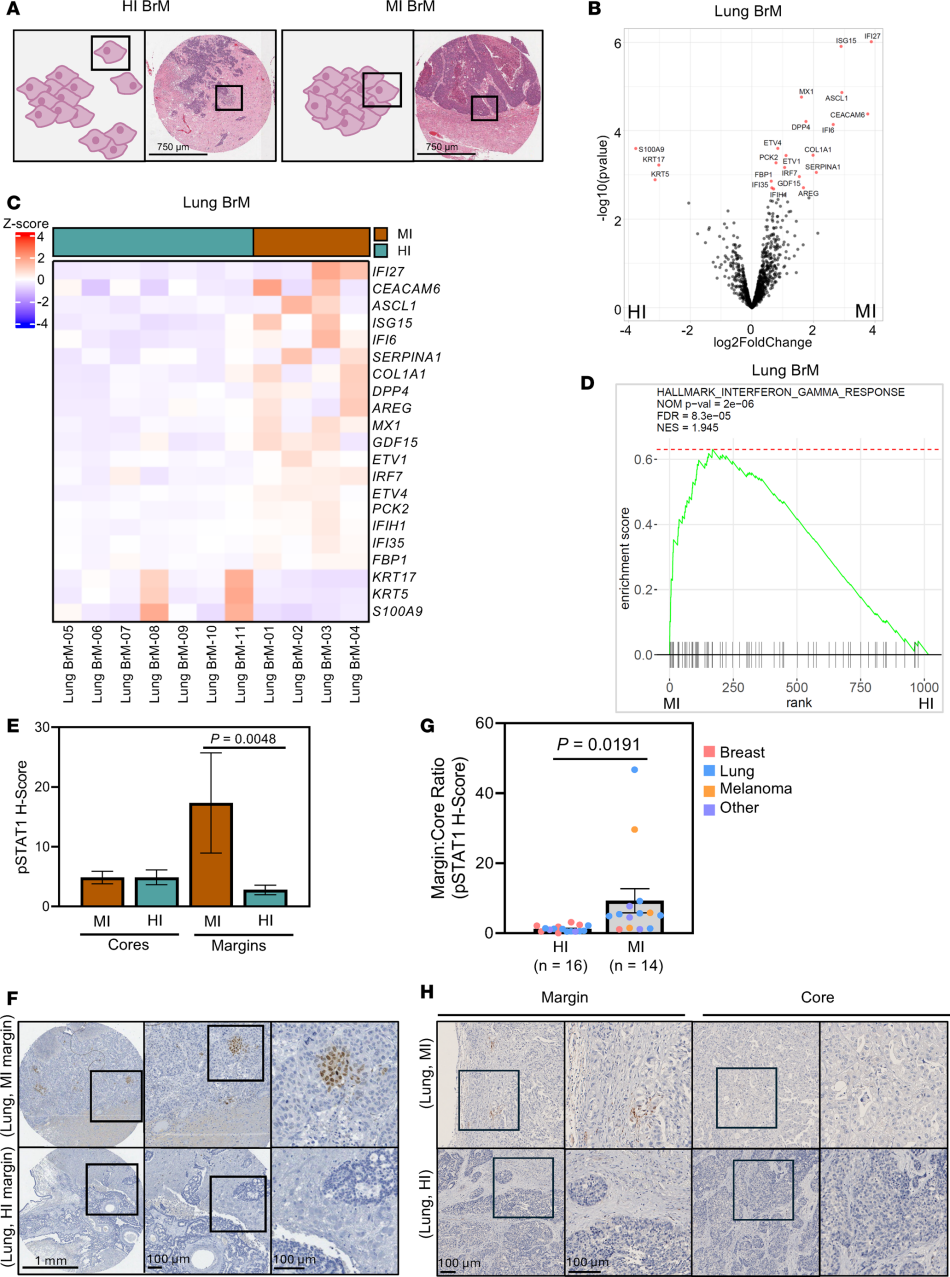
NanoString Digital Spatial Profiling reveals augmented interferon-γ signaling in MI BrMs. (**A**) Schematic depicting cancer cells selected for NanoString Digital Spatial Profiling in HI (left panels) and MI (right panels) patient BrMs. Invaded cancer cells (HI samples) or cancer cells at the brain-tumor interface (MI samples) underwent profiling. Scale bars: 750 μm. (**B**) Volcano plot illustrating differentially expressed genes (red, *n* = 21) in MI versus HI lung cancer BrM samples. Genes to the left of center are upregulated in HI samples, while genes to the right of center are upregulated in MI samples. (**C**) Heatmap depicting normalized gene expression (*z* score of log_10_-normalized gene counts) of differentially expressed genes (*P*_adj_ < 0.1; *n* = 21) in HI (blue) and MI (brown) lung cancer BrMs. (**D**) Gene set enrichment analysis enrichment plot for the interferon-γ (IFN-γ) response pathway in lung cancer BrMs. (**E**) Quantification of p-STAT1 staining (*H* score) of BrM tissue microarrays. Samples were taken at the tumor core (left; *n =* 125 MI, *n* = 258 HI) and tumor-brain margins (right; *n* = 9 MI, *n* = 28 HI). *P* values were calculated using 2-tailed Student’s *t* test. (**F**) Representative images of p-STAT1 staining (brown) in MI (top) and HI (bottom) margin samples, corresponding to data in **E**. Scale bars: 1 mm (left), 100 μm (middle and right). (**G**) Quantification of p-STAT1 staining (*H* score) in tumor cells in patient-matched core and margin regions of BrMs from any primary site (*n* = 16 HI, 14 MI). *H* scores were calculated by multiplication of staining intensity scores (0 to 3) by the percentage of positively stained tumor cells (1%–100%) for a maximum *H* score of 300. For each patient tissue, 10 regions of interest (ROIs) (200,000 μm^2^ each) were captured at the brain-tumor interface, and 10 ROIs were captured within the metastasis core (more than 1.5 mm distance from any brain-tumor interface). Margin/core *H* score ratio was calculated per sample. (**H**) Representative p-STAT1 staining (brown) in 2 lung cancer BrM samples (MI, top; HI, bottom), corresponding to data in **G**. Images of the brain-tumor interface (margin) and within the metastatic lesion (core) are shown for each sample, with high-magnification panels provided (right). Scale bars: 100 μm (all images).

While the IMC data reveal differences in immune cell frequencies between MI and HI BrMs, we next sought to characterize transcriptional differences within cancer cells that constitute MI and HI BrMs. We performed NanoString Digital Spatial Profiling using the Cancer Transcriptome Atlas to characterize transcriptional differences between cancer cells of HI and MI BrMs. We profiled 1,825 RNA targets in invading cancer cells (HI samples) or noninvading cancer cells at the brain-tumor interface (MI samples) in a cohort of breast and lung cancer BrM patient samples (Figure 2A and Supplemental Figure 2A). In lung cancer BrMs (*n* = 7 HI, *n* = 4 MI), 21 differentially expressed genes (DEGs) were identified between the 2 invasion patterns (adjusted *P* value [*P*_adj_] < 0.1) (Figure 2, B and C); the top 4 most differentially expressed genes were IFI27 (log_2_ fold change [log_2_FC] = 3.8769, *P*_adj_ = 0.0006), ISG15 (log_2_FC = 2.8957, *P*_adj_ = 0.0006), ASCL1 (log_2_FC = 2.9208, *P*_adj_ = 0.0044), and MX1 (log_2_FC = 1.6105, *P*_adj_ = 0.0044), which were elevated in MI versus HI samples (Figure 2, B and C, and Supplemental Table 1). Notably, IFI27, ISG15, and MX1 are known effectors of the interferon-γ (IFN-γ) pathway. Indeed, many of the genes that were elevated in MI versus HI lung cancer BrMs (Supplemental Table 1) are implicated in the IFN-γ pathway, such as IFI6, IRF7, IFIH1, and IFI35. Interestingly, several of the DEGs identified in the lung cohort were also identified as differentially expressed when the analyses were expanded to BrMs from both lung and breast primary sites (*n* =14 HI, 5 MI; Supplemental Figure 2, B and C, and Supplemental Table 2). Indeed, IFI27, ISG15, ASCL1, and MX1 remained the top 4 DEGs in this expanded cohort (Supplemental Figure 2, B and C, and Supplemental Table 2).

We next performed gene set enrichment analysis (GSEA) to identify differentially regulated pathways between MI and HI BrMs. Pathways found to be differentially regulated in either the lung cancer BrM–only cohort or the combined breast and lung cancer BrM cohort are shown in Supplemental Figure 2D. Interestingly, in both cohorts, the IFN-γ and IFN-α response pathways were the 2 most significantly upregulated pathways in MI versus HI samples (Figure 2D, Supplemental Figure 2E, and Supplemental Table 3), suggestive of enriched immune cell–mediated signaling in MI versus HI BrMs. As canonical IFN-γ signaling induces STAT1 activation (24), we sought to validate our transcriptomic findings though immunohistochemical (IHC) staining for phosphorylated STAT1 (p-STAT1; Y701; Supplemental Figure 2F) as a marker of IFN-γ pathway activation. We began with tissue microarrays of patient BrMs from a variety of primary sources (including lung cancer, breast cancer, melanoma) with samples taken at the tumor core or the brain-tumor margin. Although we observed no differences in p-STAT1 levels between MI and HI core samples, we observed significantly elevated p-STAT1 staining in the margins of MI versus HI BrMs (Figure 2, E and F), suggestive of a spatial gradient of STAT1 activation. For further validation, we performed IHC on whole-sample sections of surgically resected patient BrMs and quantified the margin/core ratio of p-STAT1 staining in tumor cells. We observed significantly elevated margin/core ratio of p-STAT1 signal in MI versus HI BrMs from patients with diverse primary cancers (lung, breast, melanoma, other) (Figure 2, G and H). These data reveal higher p-STAT1 positivity at the tumor margin relative to the core, and that this difference was more pronounced in MI versus HI BrMs. Such observations suggest that an elevated stromal source of IFN-γ exists in MI BrMs, consistent with an immune-hot tumor microenvironment (24).

### Spatial analyses reveal uniquely arranged lymphoid-rich regions in MI BrMs.

**Figure 3:**
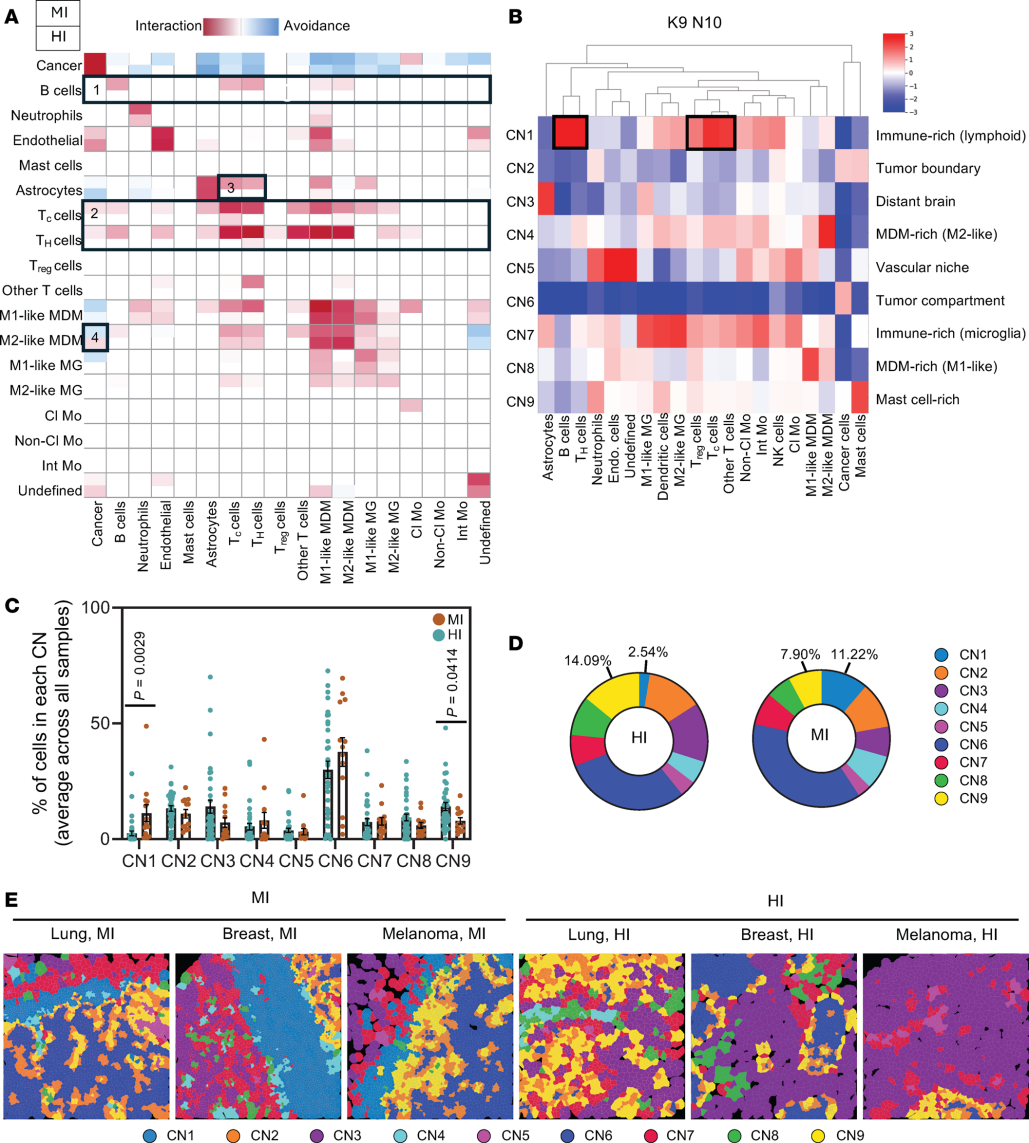
Spatial profiling reveals immune cell localization to the brain-tumor interface in MI BrMs. (**A**) Heatmap of pairwise cell-cell interaction (red) or avoidance (blue) behaviors for MI (top of square) and HI (bottom of square) BrM margin samples. Boxes highlight specific cellular associations of interest. Associations between cell types are to be read row-to-column. NK cells and dendritic cells were omitted from the heatmap owing to the absence of significant interaction or avoidance behaviors. (**B**) Heatmap of cell type distribution across 9 cellular neighborhoods (CNs) identified in BrM margins (*n* = 47 images) using *N* = 10 nearest neighbors. Boxes highlight cell populations of interest. (**C**) Bar graph depicting percentage of cells in each CN across HI (blue, *n* = 34 images) and MI (brown, *n* = 13 images) BrM margins. *P* values were calculated using 2-tailed Student’s *t* test. (**D**) Average abundance of each CN in HI (left) and MI (right) BrM margins. For each image, the percentage of cells in each CN was quantified and then averaged across all images in a category. (**E**) Representative Voronoi diagrams of CNs in MI (left) and HI (right) BrM margins from lung cancer (left), breast cancer (middle), and melanoma (right). Color code for CNs is provided.

We next sought to evaluate how the positional architecture of the tumor immune microenvironment differs between MI and HI BrMs. Cell colocalization was quantified through permutation tests to reveal interaction or avoidance behaviors between cell type pairs (Figure 3A) (25). Across the BrM margin samples from all primary sites, we found that B cells were significantly more likely to interact with other B cells, T_C_ cells, T_H_ cells, and monocyte-derived macrophages (MDMs), including M1-like MDMs and M2-like MDMs, in MI samples compared with HI samples (Figure 3A, box 1, and Supplemental Table 4). Similarly, T_C_ and T_H_ cells were significantly more likely to interact with T cells (including T_C_, T_H_, T_reg_, and Other T cells), B cells, M1-like and M2-like MDMs, and microglia (MG) in MI samples compared with HI samples (Figure 3A, box 2, and Supplemental Table 4). These data indicate that not only do MI BrMs possess more lymphoid cells than HI BrMs (Figure 1), but they are spatially organized in discrete lymphoid-rich regions rather than a diffuse lymphoid infiltrate. Additionally, astrocytes tended to interact with T_C_ and T_H_ cells in MI samples, yet this was not observed in HI samples (Figure 3A, box 3). Although direct astrocyte–T cell interactions have been best described in neurodegenerative disorders and viral infections where astrocytes contribute to T cell priming (26), the role of such interactions in brain tumors remains incompletely understood (27). In brain tumors, astrocyte–T cell interactions did not necessarily correlate with antitumor effects (28), with some evidence implicating such interactions in immune checkpoint activation and T cell inhibition (29). It has additionally been suggested that T cell–astrocyte interactions may be attributed to the act of lymphocyte transendothelial migration, where extravasating T cells encounter astrocytic end-feet (26). Indeed, our data show that T_C_ and T_H_ cells had significantly enhanced interactions with endothelial cells in MI versus HI samples (Figure 3A, box 2, and Supplemental Table 4), supporting the notion of increased T cell extravasation into MI tumors. Finally, we observed that M2-like MDMs tended to avoid cancer cells in MI samples yet favored interactions with cancer cells in HI samples (Figure 3A, box 4). This is consistent with previous work implicating direct M2-like MDM-cancer interactions in brain cancer cell proliferation and invasive growth (30).

We next explored the organization of multicellular structures within the tumor microenvironment through cellular neighborhood (CN) analyses. Two variables affect the evaluation of CNs: the total number of CNs generated (*K*), and the number of nearest neighbors evaluated for each cell that constitutes the neighborhood (*N*) (20). Using all BrM margin samples (*n* = 47 images), we first defined a constant number of CNs (*K* = 9, as in previous work) (20, 31), and altered the number of nearest neighbors evaluated (*N* = 3, 5, 10). We observed that lower *N* values (*N* = 3, 5) resulted in CNs that were largely pan-immune, with limited enrichment in specific immune cell types across CNs (Supplemental Figure 3, A and B). A higher *N* value (*N* = 10) resulted in CNs with improved discrimination between cell types (Figure 3B). The 9 CNs generated using *N* = 10 nearest neighbors represented various tissue features, including the tumor compartment (CN6) or tumor boundary (CN2), vascular niche (CN5), distant brain (CN3), immune-dense regions rich in lymphoid cells (CN1) or microglia (CN7), regions rich in M1-like (CN8) or M2-like (CN4) MDMs, and one mast cell–rich region (CN9) (Figure 3B).

When CN abundance was compared between MI and HI BrMs, 2 CNs were significantly different between the 2 growth patterns. CN9, which is enriched in mast cells, was significantly more abundant in HI versus MI BrMs (Figure 3, C and D). Mast cells have previously been found to enhance BrM cell proliferation and maintain a pro-metastatic brain microenvironment (32). Given that the mast cell–rich neighborhood (CN9) was associated with HI BrMs while mast cell frequency alone was not increased in HI BrMs (Figure 1, C–E, and Supplemental Figure 1, B and C), it is suggestive that the spatial arrangement of mast cells in BrMs is important for their pro-metastatic effects. In addition to mast cells, CN9 possessed moderate numbers of neutrophils (Figure 3B). Indeed, mast cells have been shown to recruit neutrophils to the central nervous system (CNS) (33), warranting further investigation into the role of mast cell–neutrophil interactions in HI BrMs. Secondly, CN1, which possesses abundant B cell, T_H_ cell, T_reg_, T_C_ cell, and Other T cell subsets (Figure 3B, black boxes), was significantly enriched in MI versus HI BrMs (Figure 3, C and D). Interestingly, irrespective of the *N* value used to define the CNs (*N* = 3, 5, or 10) or the number of CNs defined (*K* = 9 or 30), CNs that were significantly more abundant in MI versus HI BrMs consistently were those that were enriched in B and T cell populations (Figure 3B and Supplemental Figure 3, A–F, black boxes). This suggests that lymphoid cells are a core component of CNs that characterize MI BrMs. To localize CNs within the brain-metastatic lesions, Voronoi projections were constructed (20, 34). CN1 (Figure 3B; *K* = 9, *N* = 10) localized to the brain-tumor interface of MI BrMs, forming a lymphoid-rich band separating the metastatic cancer cells from the surrounding brain (Figure 3E). This is consistent with previous reports of peritumoral lymphocytes being abundant in a subset of patient BrMs (35, 36).

### MI BrMs possess more cytotoxically active and antigen-exposed T cells when compared with HI BrMs.

**Figure 4:**
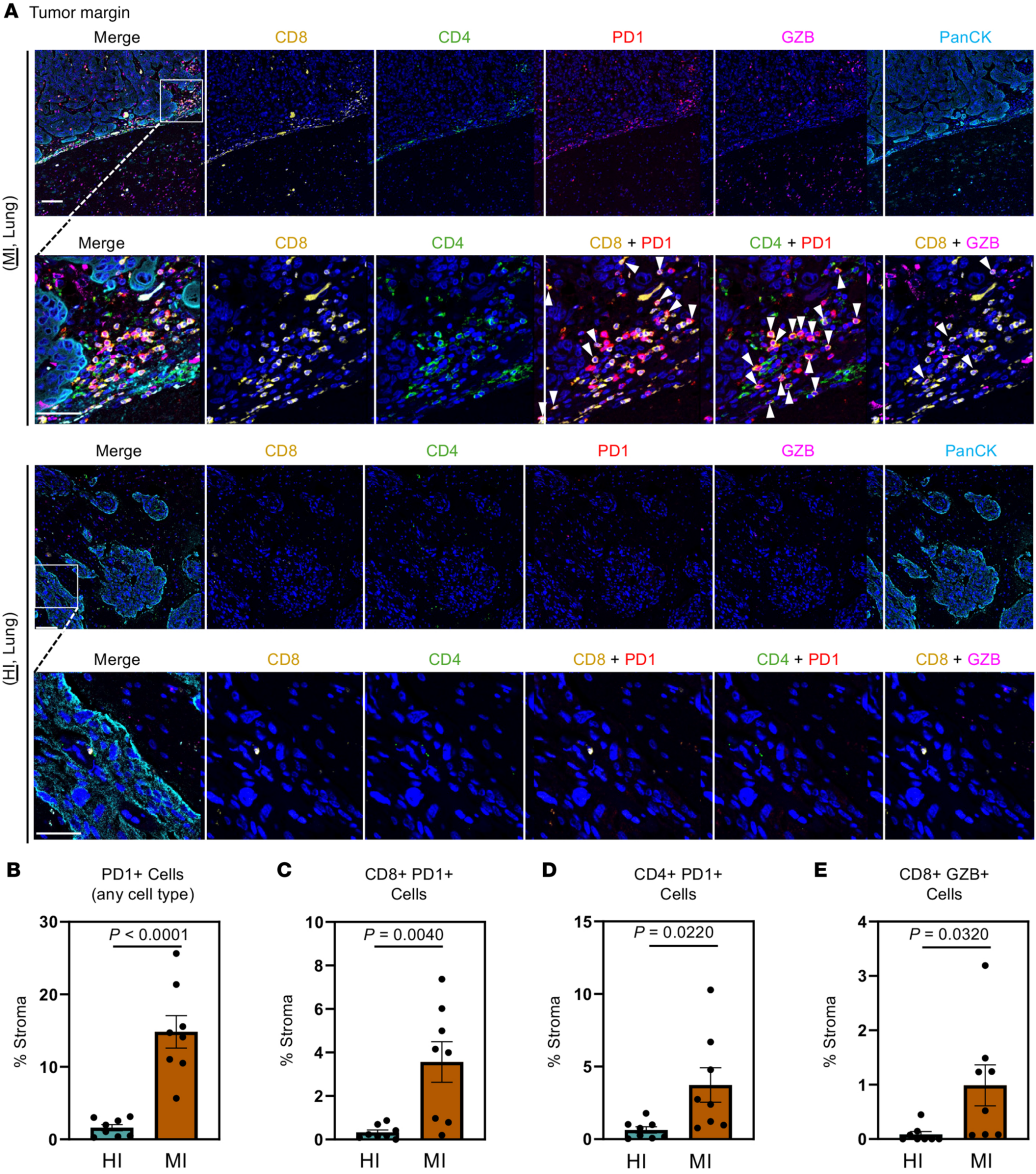
MI BrMs are characterized by granzyme B^+^ and PD-1^+^ T cells. (**A**) Representative immunohistofluorescence (IHF) staining for CD8 (yellow), CD4 (green), PD-1 (red), granzyme B (GZB; pink), and pan-cytokeratin (PanCK; cyan) in tumor margins of MI (top) and HI (bottom) lung cancer BrM patient samples. Scale bars: 100 μm (low magnification), 50 μm (high magnification). (**B**–**E**) Quantification of PD-1^+^ (**B**), CD8^+^PD-1^+^ (**C**), CD4^+^PD-1^+^ (**D**), and CD8^+^GZB^+^ (**E**) cells as a percentage of stroma (PanCK^–^ cells) in HI and MI BrM samples. *P* values were calculated using 2-tailed Student’s *t* test.

We next characterized the functional states of T cells in HI and MI BrMs. Multiplex immunohistofluorescence (IHF) staining was performed on a panel of 18 lung cancer BrMs (*n* = 9 HI, *n* = 9 MI) for CD8, CD4, PD-1, granzyme B (GZB), and pan-cytokeratin (PanCK, cancer cell marker). At the brain-tumor margins, but not the cores, there were significantly more PD-1^+^ stromal cells (PanCK^–^) in MI versus HI BrMs (Figure 4, A and B, and Supplemental Figure 4, A and B). When specifically examining T cells, we observed more CD8^+^ PD-1^+^ cells in the margins, and CD4^+^ PD-1^+^ cells in the cores and margins, of MI versus HI BrMs (Figure 4, C and D, and Supplemental Figure 4, C and D). Additionally, there were more abundant CD8^+^ GZB^+^ cells in the cores and margins of MI versus HI BrMs (Figure 4E and Supplemental Figure 4E). These data demonstrate that MI BrMs are more enriched in cytotoxically active and antigen-exposed T cells compared with HI BrMs.

### CHI3L1 contributes to tumor burden and immune-cold microenvironments in HI BrMs.

The immune-hot tumor microenvironment of MI BrMs with abundant active lymphoid cells may be conducive to response to existing therapies that leverage the immune system, such as ICIs (37). However, fewer therapeutic options exist for BrMs that are typically characterized by a lymphocyte-poor/immune-cold microenvironment, such as that observed in HI BrMs (20, 38). We therefore investigated potential mechanisms contributing to the immunosuppressive environment of HI BrMs. We previously demonstrated that chitinase 3–like-1 (CHI3L1), a secreted glycoprotein, is abundantly expressed in the tumor microenvironment of HI BrMs (8). Specifically, we determined that a subpopulation of reactive astrocytes with activated STAT3 (phosphorylated STAT3) secrete CHI3L1, which can induce cancer cell invasion into the brain parenchyma (8). However, in addition to pro-invasive functions, CHI3L1 has also been shown to induce an immunosuppressive microenvironment in both CNS and non-CNS tumors (39–45). We therefore investigated a potential role for CHI3L1 in modulating the immune microenvironment in BrMs and regulating the growth of these lesions.

**Figure 5:**
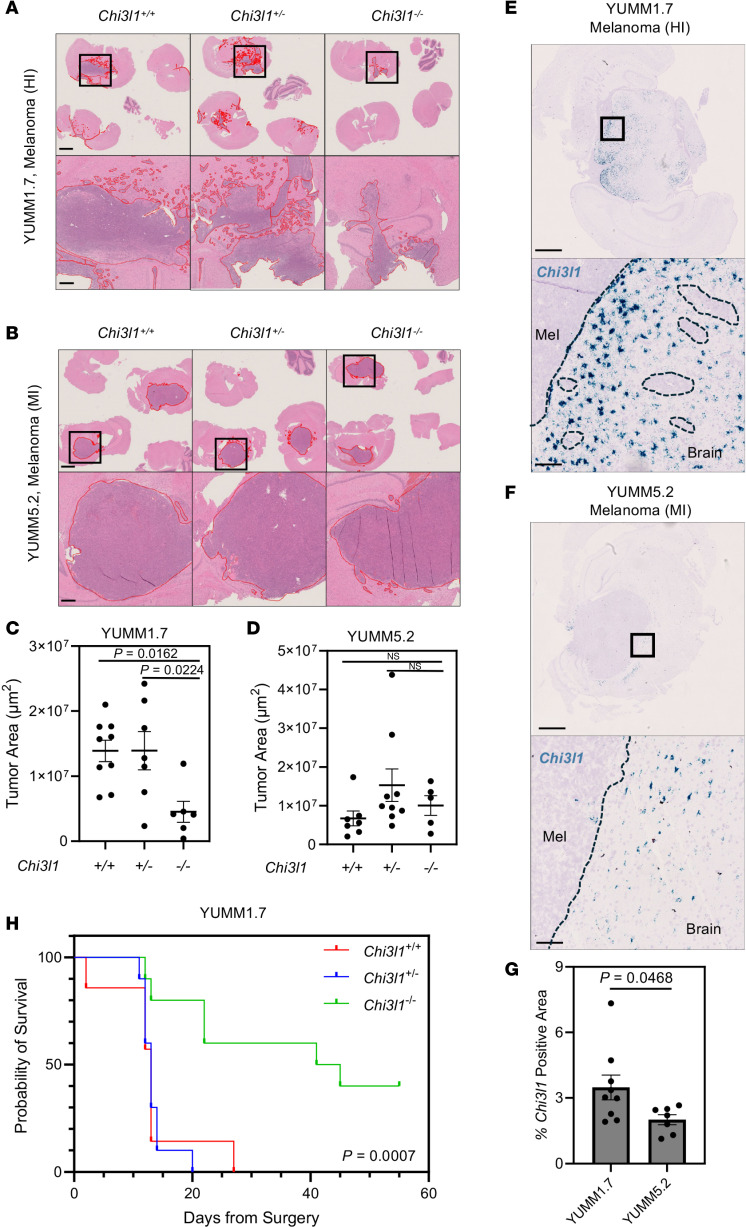
Loss of CHI3L1 decreases tumor burden in murine models of HI, but not MI, BrMs. (**A** and **B**) Representative H&E staining of brains of wild-type (+/+, left) and heterozygous (+/–, middle) and homozygous (–/–, right) *Chi3l1*-knockout mice bearing orthotopically injected HI YUMM1.7 (**A**) or MI YUMM5.2 (**B**) melanoma cells. Scale bars: 2 mm (top), 400 μm (bottom). (**C** and **D**) Quantification of tumor area across H&E images of *Chi3l1^+/+^*, *Chi3l1^+/–^*, and *Chi3l1^–/–^* mice bearing intracranially injected YUMM1.7 (**C**) or YUMM5.2 (**D**) cells. (**E** and **F**) Representative RNAscope staining for *Chi3l1* (blue) in wild-type mouse brains bearing intracranially injected YUMM1.7 (melanoma, HI) (**E**) or YUMM5.2 (melanoma, MI) (**F**) cells. Scale bars: 1 mm (top), 100 μm (bottom). (**G**) Quantification of RNAscope staining for *Chi3l1* in wild-type mouse brains bearing intracranially injected YUMM1.7 or YUMM5.2 syngeneic melanoma cells. (**H**) Kaplan-Meier survival curves for *Chi3l1*^+/+^, *Chi3l1^+/–^*, and *Chi3l1^–/–^* mice bearing intracranially injected YUMM1.7 melanoma cells. *P* value was calculated using log-rank (Mantel-Cox) test.

To do so, we used transgenic C57BL/6 mice lacking *Chi3l1*, either as full-body knockouts (*Chi3l1^–/–^*) or as heterozygotes (*Chi3l1^+/–^*) (Supplemental Figure 5, A and B) (46). We orthotopically injected syngeneic melanoma murine cells (YUMM1.7, YUMM5.2) into the brains of *Chi3l1^–/–^*, *Chi3l1^+/–^*, and wild-type (*Chi3l1^+/+^*) mice. We observed that the YUMM1.7 cells, which grow as HI lesions in the brain, showed decreased tumor burden in the *Chi3l1^–/–^* versus *Chi3l1^+/–^* or *Chi3l1^+/+^* mice, which was not observed following intracranial injection of MI YUMM5.2 melanoma cells (Figure 5, A–D). RNAscope staining for *Chi3l1* confirmed abundant *Chi3l1* in *Chi3l1^+/+^* mouse brains bearing HI YUMM1.7 tumors, with less *Chi3l1* present in the *Chi3l1^+/+^* mouse brains bearing MI YUMM5.2 tumors (Figure 5, E–G). Importantly, *Chi3l1* was expressed at low levels in the tumor compartment, and no significant difference in *Chi3l1* abundance was found in the tumor compartments of YUMM1.7 versus YUMM5.2 models (Supplemental Figure 5C). These observations are in agreement with our previously published observations demonstrating that astrocytes are the predominant source of CHI3L1 in the brain (8). Consistent with reduced tumor burden, *Chi3l1^–/–^* mice bearing intracranially injected YUMM1.7 cells displayed prolonged overall survival compared with *Chi3l1^+/–^* or *Chi3l1^+/+^* mice (Figure 5H).

We next asked whether loss of *Chi3l1* in the full-body-knockout mice affected the formation of metastases to other sites. Immunoblot analysis of lung tissue protein lysates revealed detectable Chi3l1 expression in *Chi3l1^+/+^* mice that was absent in *Chi3l1^–/–^* animals (Supplemental Figure 5B). Interestingly, tail vein injections of YUMM1.7 melanoma cells revealed no significant difference in lung metastatic burden between *Chi3l1^+/+^* and *Chi3l1^–/–^* mice (Supplemental Figure 5, D and E). These data reveal an important contribution of stromally derived CHI3L1 in the formation of CNS metastases.

**Figure 6:**
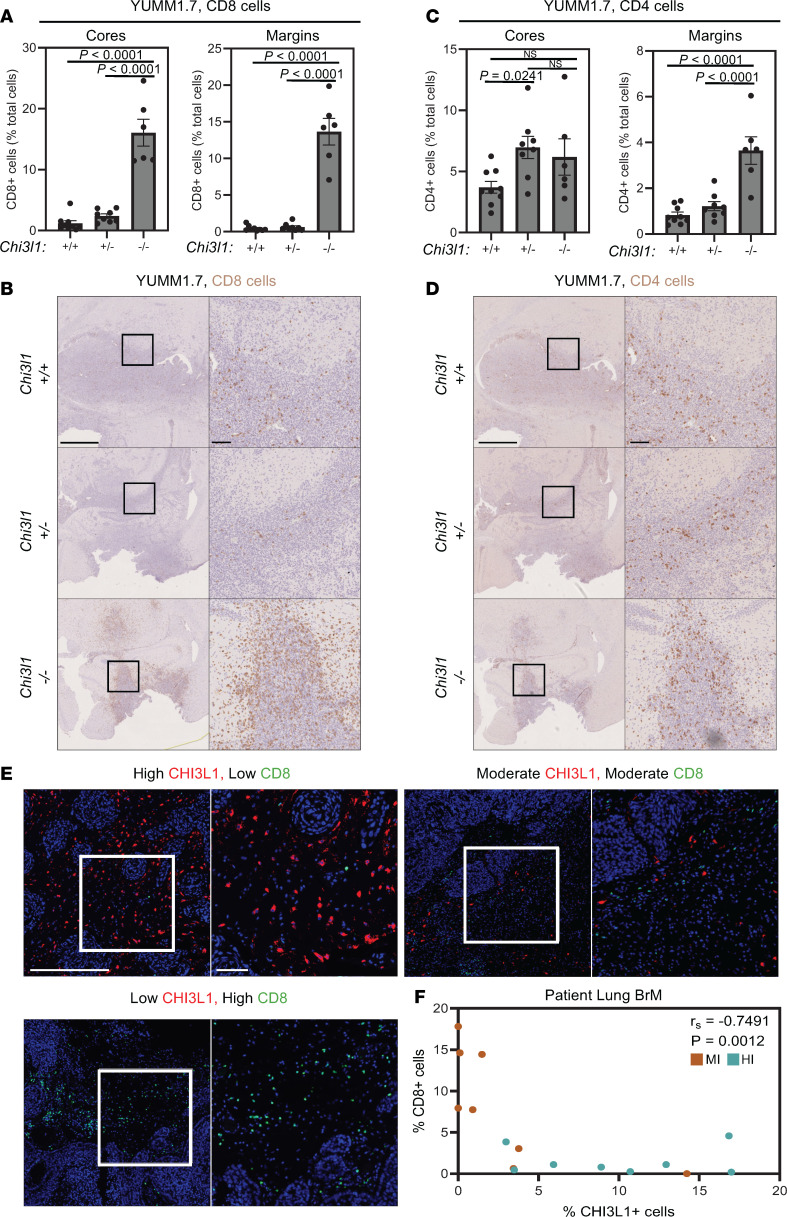
CHI3L1 levels are negatively associated with T cell infiltrate in murine models and patient BrMs. (**A**) Quantification of CD8^+^ cells in the tumor cores (left) and margins (right) of *Chi3l1^+/+^*, *Chi3l1^+/–^*, and *Chi3l1*^–/–^ mice bearing intracranially injected YUMM1.7 melanoma cells. (**B**) Representative images of immunohistochemical (IHC) staining for CD8 in YUMM1.7-derived brain lesions. Scale bars: 1 mm (left), 100 μm (right). (**C**) Quantification of CD4^+^ cells in the tumor cores (left) and margins (right) of *Chi3l1^+/+^*, *Chi3l1^+/–^*, and *Chi3l1^–/–^* mice bearing intracranially injected YUMM1.7 melanoma cells. (**D**) Representative images of IHC staining for CD4 in YUMM1.7-derived brain lesions. Scale bars: 1 mm (left), 100 μm (right). (**E**) Representative IHF staining for CHI3L1 (red) and CD8 (green) in patient lung cancer BrM samples. Scale bars: 500 μm (left images), 100 μm (right images). (**F**) Quantification of CHI3L1 and CD8 IHF staining of patient lung cancer BrM samples, corresponding to the data presented in **E**. Samples are color-coded by histopathological growth pattern (MI, brown; HI, blue). Spearman’s correlation coefficient (*r*_s_) and *P* value are provided.

To investigate how loss of *Chi3l1* affects the immune landscape of BrMs, we performed IHC staining on brain lesions derived from YUMM1.7 cells injected intracranially into *Chi3l1^+/+^*, *Chi3l1^+/–^*, and *Chi3l1^–/–^* mice. Lesions in *Chi3l1^–/–^* mice possessed significantly more CD8^+^ cells in the tumor cores and margins compared with *Chi3l1^+/+^* or *Chi3l1^+/–^* mice (Figure 6, A and B). CD4^+^ cells were also enriched at the tumor margins of YUMM1.7 lesions in *Chi3l1^–/–^* mice relative to the *Chi3l1^+/+^* or *Chi3l1^+/–^* cohorts (Figure 6, C and D). Representative locations of the margin and core regions of interest and the representative algorithm to detect CD4^+^ cells are shown (Supplemental Figure 6A). We next validated these observations in an independent, HI syngeneic lung cancer model (HKP1). We first confirmed that, like the YUMM1.7 melanoma model, wild-type mouse brains bearing HKP1 lesions possessed abundant *Chi3l1* expression in the brain parenchyma (Supplemental Figure 6B). In agreement with the results from the YUMM1.7 model, HKP1 lesions growing in *Chi3l1^–/–^* mice possessed significantly more CD8^+^ cells in the tumor cores and margins in comparison with *Chi3l1^+/+^* or *Chi3l1^+/–^* mice (Supplemental Figure 6, C and D), whereas CD4^+^ cells were only increased in the margins of *Chi3l1^–/–^* mice relative to *Chi3l1^+/+^* or *Chi3l1^+/–^* mice (Supplemental Figure 6, E and F). In both HI models, CD4^+^ cells were not enriched in the tumor cores of *Chi3l1^–/–^* mice, indicating that lack of Chi3l1 led to enhanced CD4^+^ cell recruitment but not infiltration into the lesion (Figure 6, C and D, and Supplemental Figure 6, E and F). Together, these data demonstrate a negative association between Chi3l1 levels and T cell recruitment within the tumor microenvironment of mouse BrM models.

To investigate this association in humans, we performed IHF staining for CHI3L1 and CD8 on a panel of 16 lung cancer BrM samples (*n* = 8 MI, *n* = 8 HI). We observed a negative correlation between CHI3L1^+^ cells within the brain parenchyma and infiltrating CD8^+^ cells at the brain-tumor interface, such that samples with low CHI3L1^+^ cells possessed abundant CD8^+^ cells, whereas relatively few CD8^+^ cells were evident in samples with high stromal CHI3L1 expression (Figure 6, E and F). Indeed, the samples high in CD8^+^ cells were found to be MI tumors, while those high in CHI3L1^+^ cells were largely HI tumors (Figure 6F).

### Low CHI3L1 confers improved response to immune checkpoint inhibition.

**Figure 7:**
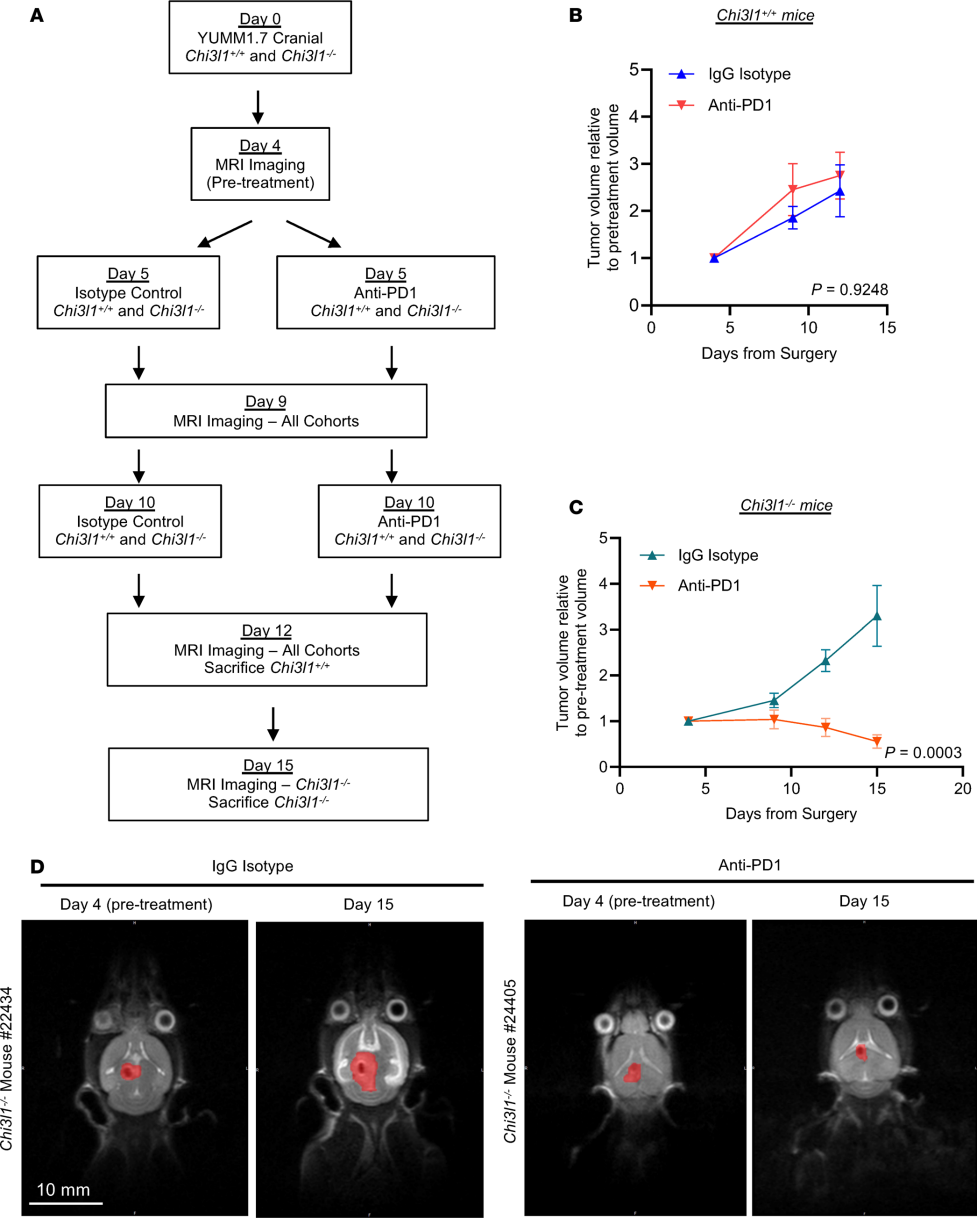
Genetic loss of Chi3l1 improves response to anti–PD-1 therapy. (**A**) Schematic illustrating experimental design. (**B** and **C**) Volume of intracranial YUMM1.7 lesions in *Chi3l1^+/+^* (**B**) and *Chi3l1^–/–^* (**C**) mice treated with IgG isotype or anti–PD-1 antibody as measured by MRI. Tumor volume is normalized to volume at day 4 (pretreatment). (**D**) Representative MRI images of intracranial YUMM1.7 lesions in *Chi3l1^–/–^* mice at pretreatment (day 4) and clinical endpoint (day 15) time points, treated with IgG isotype (left) or anti–PD-1 antibody (right). Red denotes tumor. Scale bar: 10 mm.

Multiplex IHF staining confirmed elevated CD8^+^ and CD4^+^ cells and further revealed elevated B cells, CD4^+^ FoxP3^+^ (T_reg_) cells, and CD8^+^ GZB^+^ cells in the brains of YUMM1.7 tumor–bearing *Chi3l1^–/–^* mice relative to *Chi3l1^+/+^* or *Chi3l1^+/–^* mice (Supplemental Figure 6, G–M). Given this lymphocyte-rich immune microenvironment in tumor-bearing *Chi3l1^–/–^* mice, we next asked whether loss of *Chi3l1* conferred response to ICI. We intracranially injected *Chi3l1^+/+^* and *Chi3l1^–/–^* mice with YUMM1.7 cells and treated mice with anti–PD-1 antibodies or an IgG isotype control (Figure 7A). Tumor volume was monitored over time by MRI, and cohorts were sacrificed upon reaching clinical endpoint (day 12 for all *Chi3l1^+/+^* mice, day 15 for all *Chi3l1^–/–^* mice; Figure 7A). MRI revealed that *Chi3l1^–/–^* mice treated with anti–PD-1 possessed smaller tumors at clinical endpoint compared with mice treated with IgG, while this was not observed with the *Chi3l^+/+^* cohort (Figure 7, B–D).

**Figure 8:**
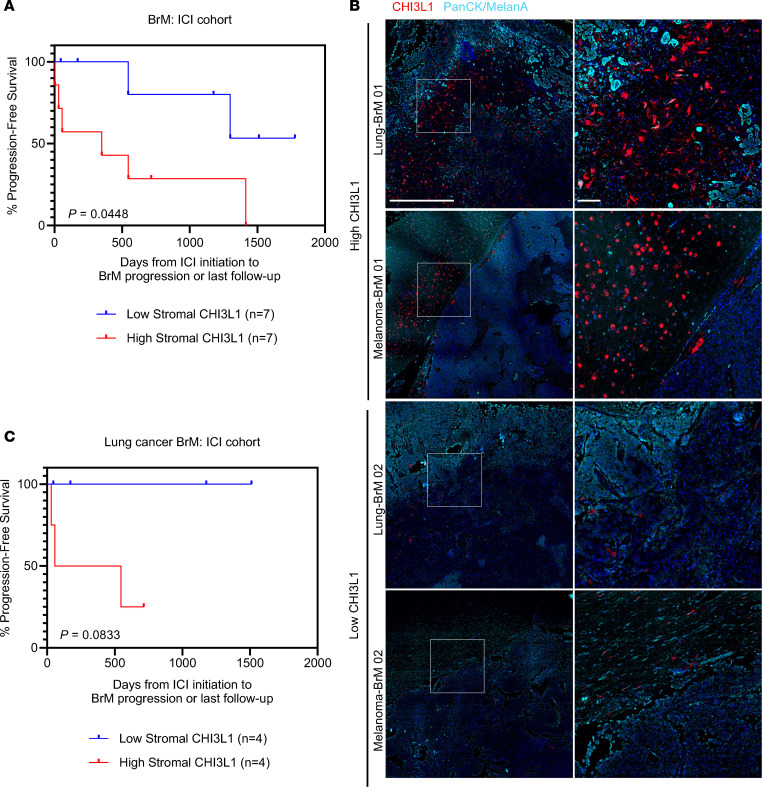
Low CHI3L1 expression in patient BrMs is associated with improved response to immune-checkpoint inhibition. (**A**) Kaplan-Meier curve showing intracranial progression-free survival of patients treated with immune-checkpoint inhibitors (ICIs). Patients were classified as having high or low stromal CHI3L1 based on median frequency of CHI3L1^+^ stromal cells at the brain-tumor margin of surgically resected samples. Patient cohorts include BrM from all primary types (*n* = 9 lung, 5 melanoma, 2 renal, 1 bladder). (**B**) Representative IHF staining for CHI3L1 (red) and cancer cells (blue, melanoma [MelanA] or pan-cytokeratin [PanCK, other primary]). Samples were classified as low or high stromal CHI3L1 based on median frequency of CHI3L1^+^ MelanA^–^/PanCK^–^ cells. (**C**) Kaplan-Meier curve showing intracranial progression-free survival of patients with lung cancer BrMs (*n* = 9, same samples were included in **A**) that were treated with ICIs. Patients were classified as having high or low stromal CHI3L1 based on median frequency of CHI3L1^+^ stromal cells at the brain-tumor margin of surgically resected samples.

We next evaluated whether stromal CHI3L1 levels could serve as a predictive biomarker for patient response to ICI. Across patients with BrMs from diverse primary sites treated with ICI (*n* = 17 patients), we observed that patients with low (below median) stromal CHI3L1 had prolonged intracranial progression-free survival compared with those with high stromal CHI3L1 (Figure 8, A and B). Sub-analysis of only the lung cancer BrM patients revealed a similar trend; however, there was a limited sample size (*n* = 9 patients), in part due to the fact that adoption of ICIs for treatment of lung cancer BrMs is quite recent (Figure 8C) (1, 3).

## Discussion

In this study, we demonstrate that histopathological growth patterns of BrM are associated with distinct tumor immune microenvironments, with MI BrMs possessing a more abundant anticancer immune infiltrate, while HI BrMs possess an immune-cold microenvironment. This is consistent with previous reports that a subset of BrMs possess an immune-hot microenvironment amid the majority being classified as immune-cold (38, 47), raising the possibility that the immune-hot samples identified in previous studies may have possessed MI tumor-brain interfaces. Indeed, one such study classified 13 of 33 BrM samples (39.4%) as being immune-hot (38), which is in alignment with our previous work describing that approximately one-third of BrMs grow in a MI pattern (4).

Many previous investigations into the BrM microenvironment have relied upon dissociated tumors, which lack spatial context. In this study, we leveraged IMC and NanoString Digital Spatial Profiling to characterize the spatial immune landscape of MI and HI BrMs. At the transcriptomic level, we reveal increased IFN signaling in cancer cells at the brain-tumor interface of MI versus HI BrMs. Through cellular interaction and neighborhood analyses, we identified a unique lymphoid-rich multicellular layer present at the brain-tumor interface in MI lesions, which was largely absent in HI lesions. These findings suggest that an immune-mediated pruning process (48) may serve to eliminate disseminated cancer cells in MI BrMs, thereby contributing to the less invasive growth pattern.

Using a combination of preclinical mouse models and human BrM samples, we identified CHI3L1 as a stromally derived secreted factor that contributes to the immune-cold microenvironment observed in HI BrMs. This builds upon our previous work demonstrating that a subpopulation of astrocytes possessing activated, phosphorylated STAT3 (p-STAT3) are abundant in HI lesions and are the predominant source of CHI3L1 in BrMs (8). p-STAT3^+^ reactive astrocytes have been implicated in enforcing an immunosuppressive microenvironment in BrM, through release of factors such as vascular endothelial growth factor-A (VEGF-A), lipocalin-2, and tissue inhibitor of metalloproteinases-1 (TIMP1) (21, 49). The data reported herein demonstrate an immunosuppressive role for CHI3L1 in BrMs, supporting the addition of CHI3L1 to the list of p-STAT3^+^ reactive astrocyte–derived immunosuppressive proteins in the BrM microenvironment.

CHI3L1 has previously been reported as enforcing immune suppression in a variety of cancer types, including gastric cancer, breast cancer, hepatocellular carcinoma, lymphoma, melanoma lung metastases, and glioblastoma (39–45). This work is the first, to our knowledge, to demonstrate the immunosuppressive effects of CHI3L1 in the context of BrM; however, the exact mechanism(s) through which CHI3L1 exerts these effects remains an important area of future research. The specific mechanism for CHI3L1-mediated immunosuppression is varied across and within tumor types, with reports of CHI3L1-induced neutrophil-mediated T cell exclusion in breast cancer (43) versus shifting of macrophage populations from anti-tumorigenic M1-like toward pro-tumorigenic M2-like reported in breast cancer, gastric cancer, and glioblastoma (39, 44). It has also been shown that CHI3L1 can suppress macrophage-mediated phagocytosis in the context of melanoma lung metastases (50). Our previous data indicated that a small percentage of the immune infiltrate is composed of neutrophils, with macrophages representing the dominant innate immune cell type in BrMs (20). However, future experiments will be required to determine whether either of these innate immune cells is responsible for CHI3L1-mediated immunosuppression. In addition to its effects on innate immune cells, CHI3L1 has also been implicated in regulating T cell adaptive immunity (45, 51). Chi3l1 was found to suppress T_H_ cell expression of anti-tumorigenic genes (*Ifng*, *Ctse*, *Cxcr2*, and *Tnfsf10*) as well as various effector molecules in T_C_ cells (T-bet, perforin, and granzyme B), implicating Chi3l1 as a negative regulator of T_H_ and T_C_ cell functions (51). Moreover, CHI3L1 has been shown to impair the expression of T cell costimulatory molecules (ICOS, ICOSL, CD28) and augment the expression of immune checkpoint proteins (B7-1 and CTLA4) in the lungs of mice bearing melanoma metastases (45). Further investigations would be required to determine whether similar mechanisms are present in the context of BrM.

Clinical trials continue to show only a subset of BrM patients responding to ICIs (12–16), prompting interest in identifying clinical and biological features that characterize these patients. There has additionally been growing argument for avoiding unnecessary treatment with ICIs to spare patients the risk of serious adverse events (14, 52), which have been reported in up to 52% of patients with BrMs treated with ICI (14). This has led to studies identifying unique T cell populations (38), cancer cell markers (13), gene mutations (53), epigenetic modifications (54), or circulating factors (49) that may serve as clinically relevant biomarkers for BrM patient stratification for ICI treatment. Our observed inverse association between stromal CHI3L1 levels in surgically resected BrMs and intracranial response to ICI treatment supports further investigation into the use of CHI3L1 as a potential biomarker to identify patients who are more likely to be refractory to ICI-based therapies. One possibility is through measuring circulating levels of CHI3L1, which consistently correlate with poor outcomes in various cancer types, including brain cancers (55–61). Importantly, circulating CHI3L1 is predictive of clinical benefit of ICIs for patients with metastatic pancreatic cancer (58), underscoring the relevance of investigating a possible similar predictive role in BrM. Our observation that loss of CHI3L1 increases T cell abundance, including the presence of activated (GZB^+^) CD8^+^ T cells, raises the possibility that CHI3L1 inhibition may result in a microenvironment that is more conducive to ICI response. Indeed, this has been observed in murine models of breast cancer and melanoma lung metastases, whereby an anti-CHI3L1 neutralizing antibody conferred increased T cell infiltration and improved response to immunotherapy (43, 62). In a murine model of glioblastoma, a mimetic peptide interfering with CHI3L1 receptor binding has been shown to disrupt CHI3L1-mediated macrophage reprogramming and inhibit tumor progression (44). Bispecific neutralizing antibodies against CHI3L1/PD-1 have been shown to have a synergistic antitumor effect mediated by enhanced CD8^+^ T cell activity, warranting further investigation into the clinical role of dual-targeting antibodies in immunotherapy (62). Inhibition of STAT3, the main transcriptional factor regulating CHI3L1 (63), may also serve to reduce CHI3L1-mediated immunosuppression in BrM. STAT3 inhibition in reactive astrocytes via silibinin (21, 64) has shown efficacy in reducing tumor burden in models of HI BrM (8) and has demonstrated intracranial clinical benefit in a subset of patients with BrMs (21). Moreover, a recent study has found that silibinin treatment in combination with ICI in a radiotherapy-treated murine model of BrM indeed increased the antitumor response, with increased markers of cytotoxic activity (49). Our data presented here demonstrating the immunosuppressive effects of the STAT3 target CHI3L1 provide additional credence to the notion of combining silibinin and ICI in the treatment of BrM. While targeting CHI3L1 represents an exciting therapeutic approach to treat non-CNS cancers, more information regarding the mechanisms through which CHI3L1 enhances the invasive growth of BrMs and the ability of the therapeutics mentioned above to cross the blood-brain barrier is required before the best strategy can be selected for the treatment of BrM.

## Methods

### Sex as a biological variable

The study used surgically resected BrM samples from both male and female patients. Male and female mice were used in in vivo experiments, with sex balanced between treatment arms where applicable. Data are presented in aggregate across both sexes.

### Clinical specimens and data

#### Growth pattern classification.

Surgically resected BrMs were stained by H&E and evaluated by a neuropathologist. The criteria for histopathological growth pattern classification of BrMs have been previously described (4).

#### Immune checkpoint inhibition cohort and progression-free survival analyses.

Patient clinical charts were reviewed by clinicians and evaluated for treatment with an ICI. Patients receiving at least 2 cycles of ICI following BrM surgical resection were included. Imaging reports (MRI or CT) were evaluated for evidence of intracranial tumor progression (development of leptomeningeal disease, local recurrence, or new metastatic lesion).

### NanoString GeoMx Digital Spatial Profiling

#### Sample preparation.

A tissue microarray (TMA) was constructed from FFPE surgically resected BrM tissue. Selected tissue blocks captured the interface between the BrM lesion and the adjacent brain. Unstained slides from this TMA were sent to NanoString Technologies Inc. Once received, slides were stained with PanCK AE1/AE3/PCK26 antibody (Roche, 760-2595). Via teleconference, segmentation was performed and geometric areas comprising cancer cells at the tumor-brain interface were selected for subsequent profiling.

#### Target matrix processing.

As in standard NanoString workflow, 1–5 probes were used to measure the expression of each target gene (*n* = 1,825) and 88 spike-in probes as negative controls. The geometric mean of each target and negative control was calculated per region of interest (ROI). The limit of quantification (LOQ) was calculated, per ROI, as 2 standard deviations above the geometric mean of the negative controls. Targets with no values across ROIs above the LOQ were excluded from the analysis (65). One ROI was excluded from the analysis because of a low total probe count.

#### Differential expression analysis.

Differential gene expression analysis between HI and MI conditions was performed for lung and breast samples using the DESeq2 R package (version 1.34.0) (66). The Benjamini-Hochberg procedure (pAdjustMethod = “BH”) was used to control for multiple testing (67). The geometric mean matrix was rounded so it could be used as input for DESeq2. The *z* scores of the counts, log_10_-normalized by library size, of genes with adjusted *P* value less than 0.1 were visualized as heatmaps (68).

#### Gene set enrichment analysis.

Gene set enrichment analysis (GSEA) was performed using the log fold change between the HI (baseline) and MI conditions of all the genes (*n* = 1,627) that were measured. The hallmark gene set (*n* = 50, version h.all.v2023.2.Hs) from the Molecular Signatures Database (MSigDB) and the FGSEA R package (version 1.20.0) were used for this analysis (69–71).

### Immunohistochemistry

Tissues were fixed in 10% formalin for 24 hours and paraffin-embedded, and 4 μm sections were cut. IHC was performed using the Ventana Benchmark Ultra Autostainer for the p-STAT1 antibody (Cell Signaling, 9167; Tyr701, 1:200). For the following antibodies, IHC was performed manually: CD4 (Cell Signaling, 25229; 1:50) and CD8 (Cell Signaling, 98941; 1:400). Sections underwent successive incubations in xylene and decreasing concentrations of ethanol. Antigen retrieval was performed in Tris-based buffer (Vector Laboratories, H3301) for 10 minutes using a pressure cooker. Sections were incubated in 3% hydrogen peroxide for 10 minutes and subsequently blocked in casein-based buffer (Vector Laboratories, SP-5020) at room temperature. Primary antibodies were diluted in 2% BSA and incubated overnight at 4°C. Slides were washed in 2% BSA, and SignalStain Boost secondary antibody (Cell Signaling, rabbit, 8114S) was applied for 30 minutes at room temperature. The SignalStain DAB substrate kit (Cell Signaling, 8059) was used for detection, followed by counterstaining with hematoxylin and subsequent dehydration and mounting. Slides were scanned at ×20 with a NanoZoomer S210 slide scanner (Hamamatsu).

### Immunohistofluorescence

Immunohistofluorescence (IHF) staining was performed using the Opal 6-Plex detection kit (Akoya Biosciences), as previously described (20). The following antibodies were used: CD8 (Dako, M7103; 1:50), CD4 (Abcam, 133616; 1:100), PD-1 (Cell Signaling, 86163; 1:50), GZB (Roche, 760-4283; 1:5), PanCK (Roche, 760-2595; 1:10), MelanA (Sigma-Aldrich, M6570; 1:50), and CHI3L1 (Cell Signaling, 47066; 1:400). For 6-color IHF multiplex, images were acquired using a Zeiss 710 inverted confocal microscope with ×20 objective, and images were tiled (2 × 2). A minimum of 3 images were captured per region (core, margin) per sample. For the 2-color IHF staining, slides were scanned at ×20 on a Zeiss AxioScan.

### RNAscope

RNAscope was performed on FFPE tissue using the *Chi3l1* probe (Advanced Cell Diagnostics, 449621) as previously described (8). Slides were scanned at ×40 with a NanoZoomer slide scanner.

### Image analysis

IHC, IHF, and RNAscope image analysis was performed using HALO image analysis software (version 3.5, Indica Labs).

For p-STAT1 IHC staining, 10 ROIs (2 × 10^5^ μm^2^ each) were annotated at the brain-tumor interface (“margin”), and 10 ROIs were annotated within the metastasis (“core”), a minimum 1.5 mm distance away from any brain-tumor interface. Staining intensity scores were calculated on a scale of 0 (absent) to 3 (strong). *H* scores were generated by multiplication of the staining intensity scores (0 to 3) by the percentage of cells with positive staining (0%–100%), for a maximum possible *H* score of 300. The margin/core *H* score ratio was calculated for each patient sample.

For CD8/CD4 IHC staining, 7 ROIs (200,000 μm^2^ each) were annotated at the brain-tumor interface (“margin”), and 8 ROIs were annotated within the tumor (“core”). Positively stained cells were quantified.

For T cell multiplex IHF, cell types were classified based on the following marker expression requirements: CD8^+^ T cells (CD8^+^CD4^–^PanCK^–^), CD4^+^ T cells (CD8^–^CD4^+^PanCK^–^), cancer cells (CD8^–^CD4^–^PanCK^+^). Cell types were quantified and averaged across image replicates.

For CHI3L1 IHF staining, images were annotated at the brain-tumor interface to generate margin ROIs (500 μm depth into the brain), with a total minimum area of 5 × 10^6^ μm^2^ per slide. CHI3L1^+^ stromal cells were defined as CHI3L1^+^ PanCK^–^/MelanA^–^.

For RNAscope, images were annotated at the brain-tumor interface to generate margin ROIs (500 μm depth into the brain), and *Chi3l1^+^* pixels were quantified. Percentage positive area was calculated. For analysis of the tumor compartment, tumor cells were annotated, and *Chi3l1^+^* pixels were quantified within the annotated region. Percentage positive area was calculated.

H&E image analysis for tumor burden was performed using QuPath software (version 0.3.2) (72).

### Imaging mass cytometry

The imaging mass cytometry (IMC) images used in this paper have been previously published (20). Briefly, surgically resected BrM samples were formalin-fixed, paraffin-embedded, and assembled into TMAs (1.5 mm cores). TMAs underwent immunostaining, IMC, cell segmentation, and lineage assignment as previously described (20).

#### Cell-cell pairwise interaction analysis.

Significant pairwise interaction or avoidance behaviors were identified through permutation tests of single-cell interactions, as previously described (25). Briefly, the locations of each cell type within each IMC image were randomly permutated 50,000 times, maintaining the original frequency of cell types. For each permutation, the frequency of interactions between any 2 cell types was quantified and compared with the actual arrangement, resulting in interaction and avoidance scores ranging from 0 to 50,000 for each image. Scores were aggregated to construct interaction and avoidance distributions for each pairwise cell type comparison, using a score cutoff of 25,000. Cells within a 6-pixel radius (6 μm) were defined as interacting. Interaction and avoidance distributions were compared between HI and MI groups, and *P* values less than 0.05 were defined as significant.

#### Cellular neighborhood analysis.

Methodology for identifying cellular neighborhoods (CNs) has been previously described (20). Briefly, neighbor windows were computed based on the number (*N*) of nearest cells to a given cell and then clustered using the MiniBatchKMeans clustering algorithm version 1.1.2 (scikit-learn, a software machine learning library for Python). Each cell was assigned to a CN based on its neighbor window. CN prevalence for each sample was normalized such that the sum of CN prevalence was 100% for each sample.

### Cell culture

YUMM1.7 cells were obtained from the ATCC. YUMM5.2 and HKP1 cells were provided by Marcus Bosenberg (Yale University, New Haven, Connecticut, USA) and Vivek Mittal (Weill Cornell Medicine, New York, New York, USA), respectively. HKP1 cells were cultured in DMEM supplemented with 10% FBS. YUMM1.7 and YUMM5.2 cells were cultured in DMEM:F12 medium supplemented with 10% FBS and 5% nonessential amino acids.

### Mouse experiments

Chi3l1-knockout mice. Chi3l1–/– mice were obtained in-house (46). Chi311–/– mice were crossed with C57BL/6J mice (The Jackson Laboratory) to generate heterozygous Chi3l1+/– mice. Breeding pairs of Chi3l1+/– mice were set up to generate offspring of all 3 genotypes. Tail DNA was collected and underwent PCR genotyping using the following primers: Chi3l1 pair, ACCTAGTGTCCTTCTGGCCTTGG and CCAGAGACAGGAACTGGCTGAG; neomycin pair, TGCTCCTGCCGAGAAAGTAT and AGCTGGCCCTTAATTTGGTT. Genotypes were as follows: Chi3l1+/+ mice: absent neomycin, present Chi3l1; Chi3l1+/– mice: present neomycin, present Chi3l1; Chi3l1–/– mice: present neomycin, absent Chi3l1.

Orthotopic injection of cancer cells. Cells (1 × 105) suspended in 5 μL of PBS were intracranially injected 2 mm to the right and 1 mm rostral to the confluence of sinuses, using a guide screw method as previously described (4, 73). Experiments were terminated and all mice were euthanized when the first mouse in any arm reached clinical endpoint, with the exception of survival assays, whereby mice were individually euthanized upon reaching individual clinical endpoint as determined by blinded animal health technicians.

Treatment with anti–PD-1. Chi3l1+/+ (n = 17) and Chi3l1–/– (n = 16) mice were intracranially injected with 1 × 105 YUMM1.7 melanoma cells, as described above. Mice were imaged by MRI (M2 Compact MRI, Aspect Imaging) on day 4 after injection, and mice were allocated into treatment arms based on pretreatment tumor volume to ensure minimal variation between cohorts. Mice received intraperitoneal injection of anti–PD-1 (10 mg/kg) or IgG2a rat isotype control (10 mg/kg) on days 5 and 10. Mice were regularly imaged by MRI to monitor tumor volume over time (days 9, 12, and 15), using the following imaging parameters: T2-weighted fast spin echo with echo time/repetition time = 4,000 milliseconds/77.6 milliseconds for about 6 minutes per mouse, with slice thickness of 1.00 mm and spacing as 1.10 mm and echo-train length of 12. MRI images were analyzed using VivoQuant software (v2020, Invicro). Tumor volumes were normalized to pretreatment volume for each individual mouse.

Tail vein injections. YUMM1.7 cells (2 × 105) were injected into the lateral tail veins of Chi3l1+/+ (n = 6) and Chi3l1–/– (n = 5) mice. At clinical endpoint, lungs were harvested and processed into FFPE blocks and underwent H&E staining. Regions of tumor within the lungs were annotated, and lung-metastatic area was quantified.

### Immunoblotting

*Chi3l1^+/+^*, *Chi3l1^+/–^*, and *Chi3l1^–/–^* mice were euthanized, and lungs were extracted and snap-frozen in liquid nitrogen. Tissue was homogenized using a mortar and pestle and underwent cell lysis. Lysates were resolved by SDS–polyacrylamide gel electrophoresis and transferred to Immobilon FL PVDF membranes (Millipore, IPFL00010). Membranes were blocked using Intercept Blocking Buffer (LI-COR Biosciences, 927-600001) and incubated with antibodies against Chi3l1 (Invitrogen, 81355; 1:500) and α-tubulin (Sigma-Aldrich, T9026; 1:10,000) overnight at 4°C. Fluorophore-conjugated secondary antibodies (IRDye 800 anti-rabbit, IRDye 680 anti-mouse, LI-COR Biosciences) were applied for 1 hour at room temperature. Bands were visualized using the LI-COR Odyssey Imaging System.

### Statistics

Statistical tests were performed using GraphPad Prism 10. The specific statistical tests used for each analysis are indicated in the corresponding figure legend. Briefly, when 2 conditions were compared, a 2-tailed unpaired Student’s *t* test was used. When more than 2 conditions were compared, 1-way ANOVA tests were used. *P* values less than 0.05 were considered significant. Survival data are presented as Kaplan-Meier curves, with analyses performed using the log-rank (Mantel-Cox) test.

### Study approval

All patient samples were obtained after informed, written consent and were deidentified. Studies were performed in compliance with institutional review boards of McGill University and the Montreal Neurological Institute-Hospital (IRB 2018-4150) and in accordance with the 1996 Declaration of Helsinki. All animal work was performed in compliance with animal use protocols approved by the McGill University Animal Resources Centre (2001-4830) and with guidelines established by the Canadian Council on Animal Care.

### Data availability

Values for all data points in graphs are reported in the Supporting Data Values file. The code for the NanoString GeoMx Digital Spatial Profiling analysis, including target matrix processing, differential expression analysis, and GSEA, is available at https://www.ncbi.nlm.nih.gov/geo/query/acc.cgi?acc=GSE303531

## Author contributions

Research studies were designed by SMM, LAW, and PMS. Experiments were conducted and data collected by SMM, MD, YAH, MGA, NK, EP, PF, and YW. Data analysis was performed by SMM, EK, AHC, MWY, YAH, MR, BL, BF, AN, and LAW. Reagents were provided by JAE, CGL, KP, and MCG. The manuscript was written by SMM and PMS. All authors reviewed and edited the manuscript. The study was supervised by MP, YR, HN, KP, MCG, DFQ, LAW, and PMS.

## Conflict of interest

JAE is a cofounder of Elkurt Therapeutics and Sakonnet Biomedical, which develop inhibitors of 18-glycosyl hydrolases as therapeutics. JAE and CGL have composition of matter and use patents relating to antibodies against CHI3L1. CGL serves as a consultant for siRNAgen Inc., which develops RNA therapeutics.

## Funding support

The following entities provided funding.

- McGill MD/PhD Program (to SMM).
- Vanier Canada Graduate Scholarship (to SMM).
- Canadian Institutes of Health Research (CIHR PJT-175066 to PMS).

## Supplementary Material

Supplemental data

Unedited blot and gel images

Supporting data values

## Version 1 05/22/2026

Electronic publication
