# Supplementary material for: Distinct immune landscapes characterize highly versus minimally invasive brain metastases
Source: JCI Insight. 2026 May 22;11(10):e199498. doi: 10.1172/jci.insight.199498 (PMC13232719; doi:10.1172/jci.insight.199498)
Supplement: Supplemental data [file jciinsight-11-199498-s137.pdf]

a

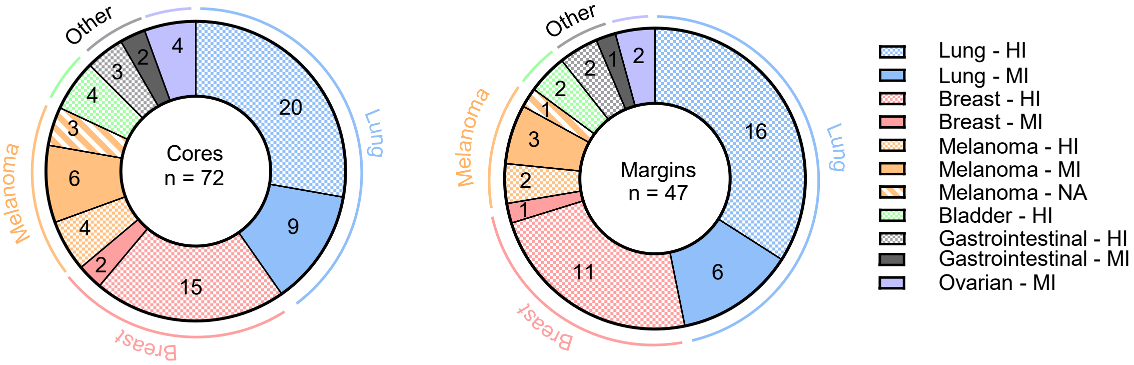

Maritan *et al.* Extended Data Fig. 1

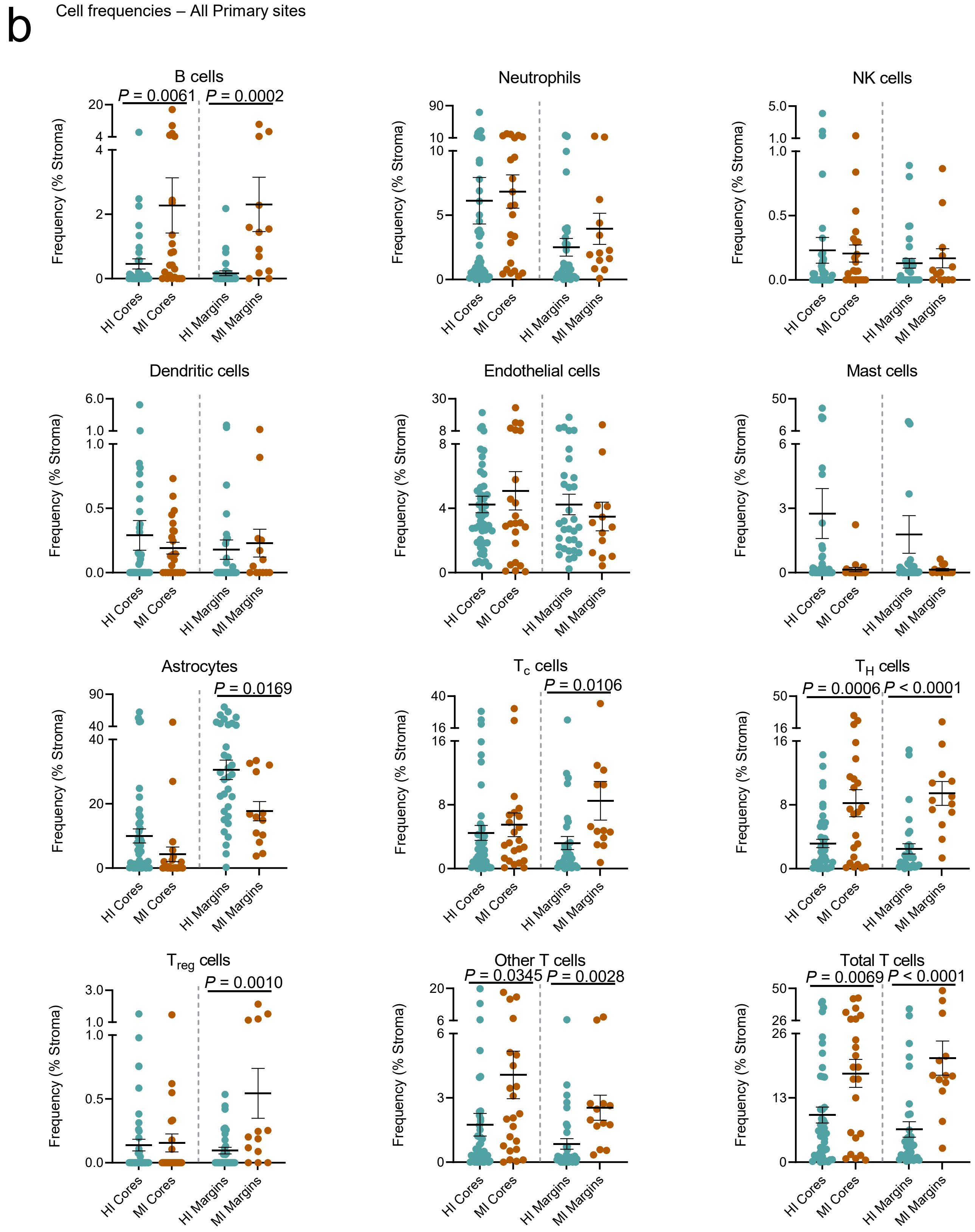

Maritan *et al.* Extended Data Fig. 1, Continued

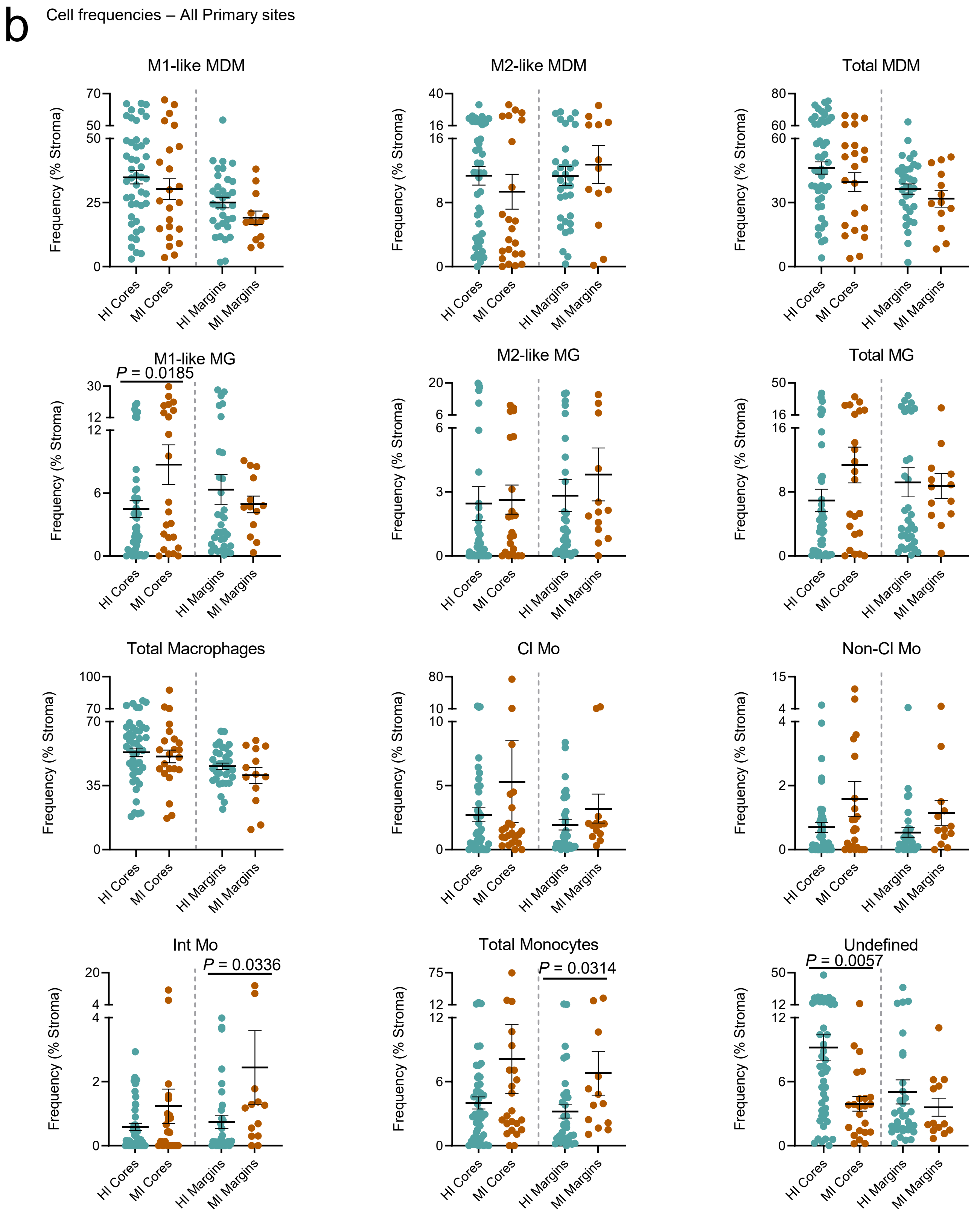

Maritan *et al.* Extended Data Fig. 1, Continued

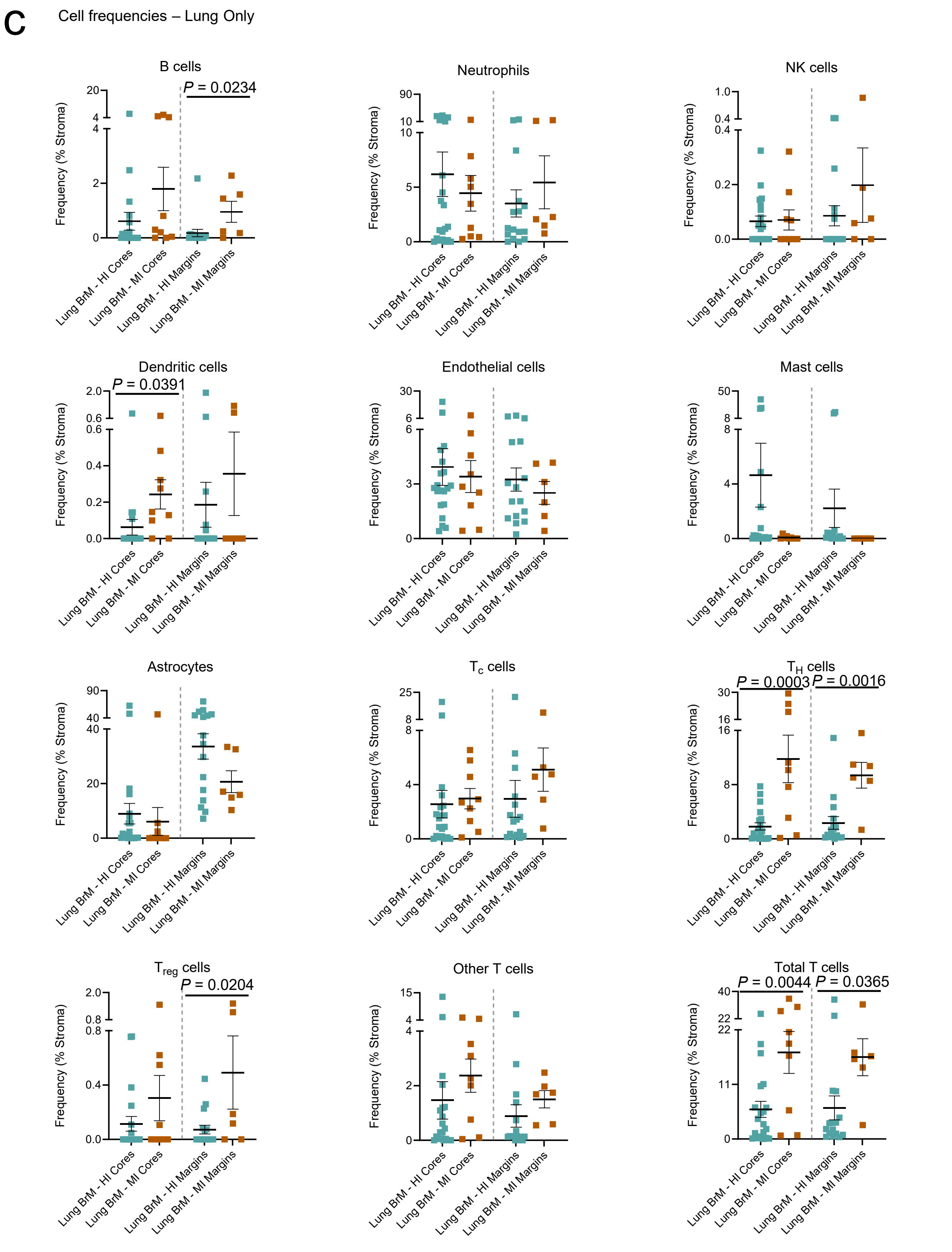

Maritan *et al.* Extended Data Fig. 1, Continued

C Cell frequencies – Lung Only

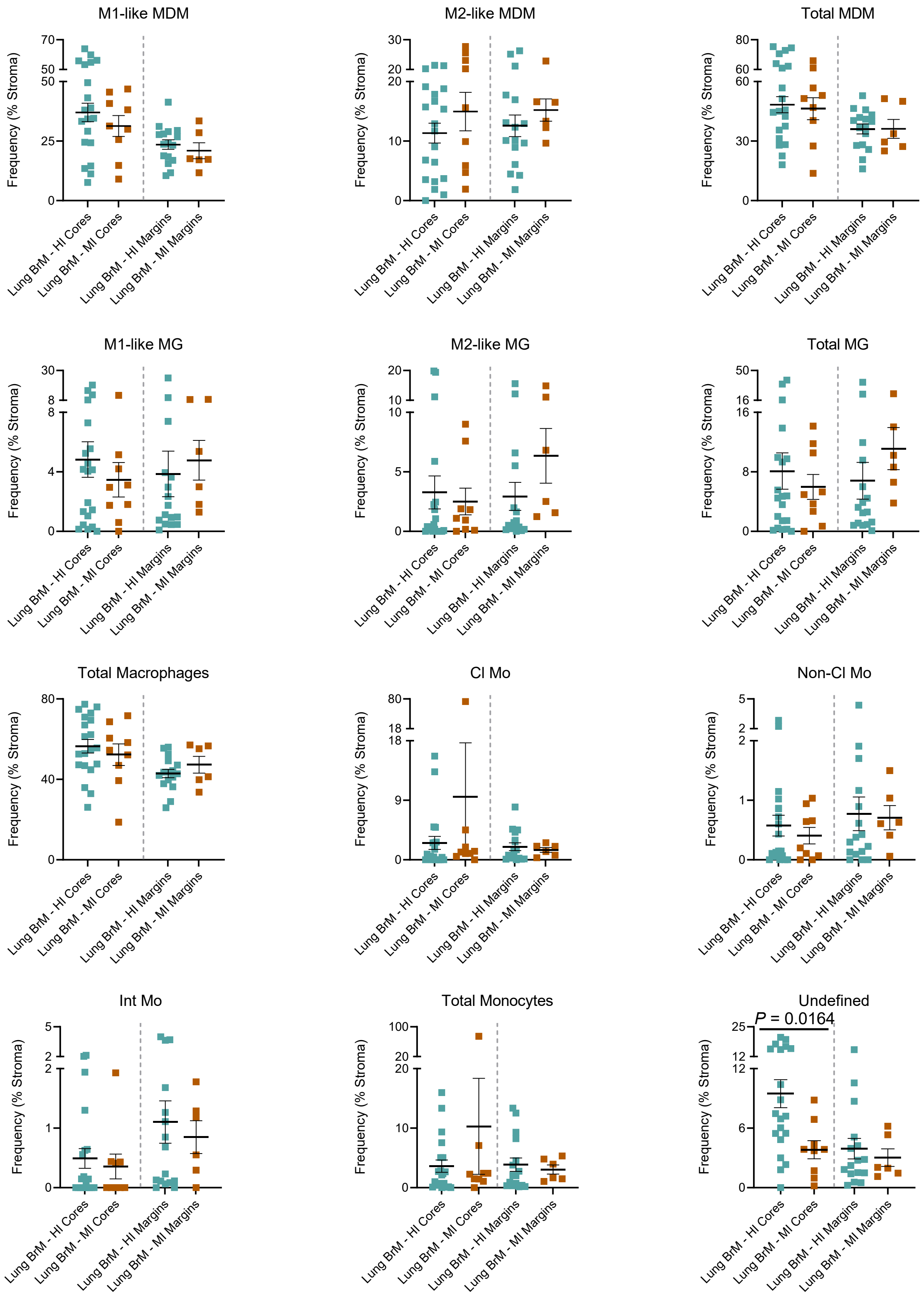

Maritan *et al.* Extended Data Fig. 1, Continued

Extended Data Fig. 1. Cell frequencies in highly and minimally invasive brain metastases as determined by imaging mass cytometry. a, Distribution of human brain metastasis IMC images across primary sites and sampling regions (cores, *left*; margins, *right*). **b, c,** Cell frequency as a proportion of stroma in core and margin samples of HI and MI BrM from all primary sites (**b**) or lung cancer only (**c**). P values were calculated using Student's t test. P values less than 0.05 are shown.

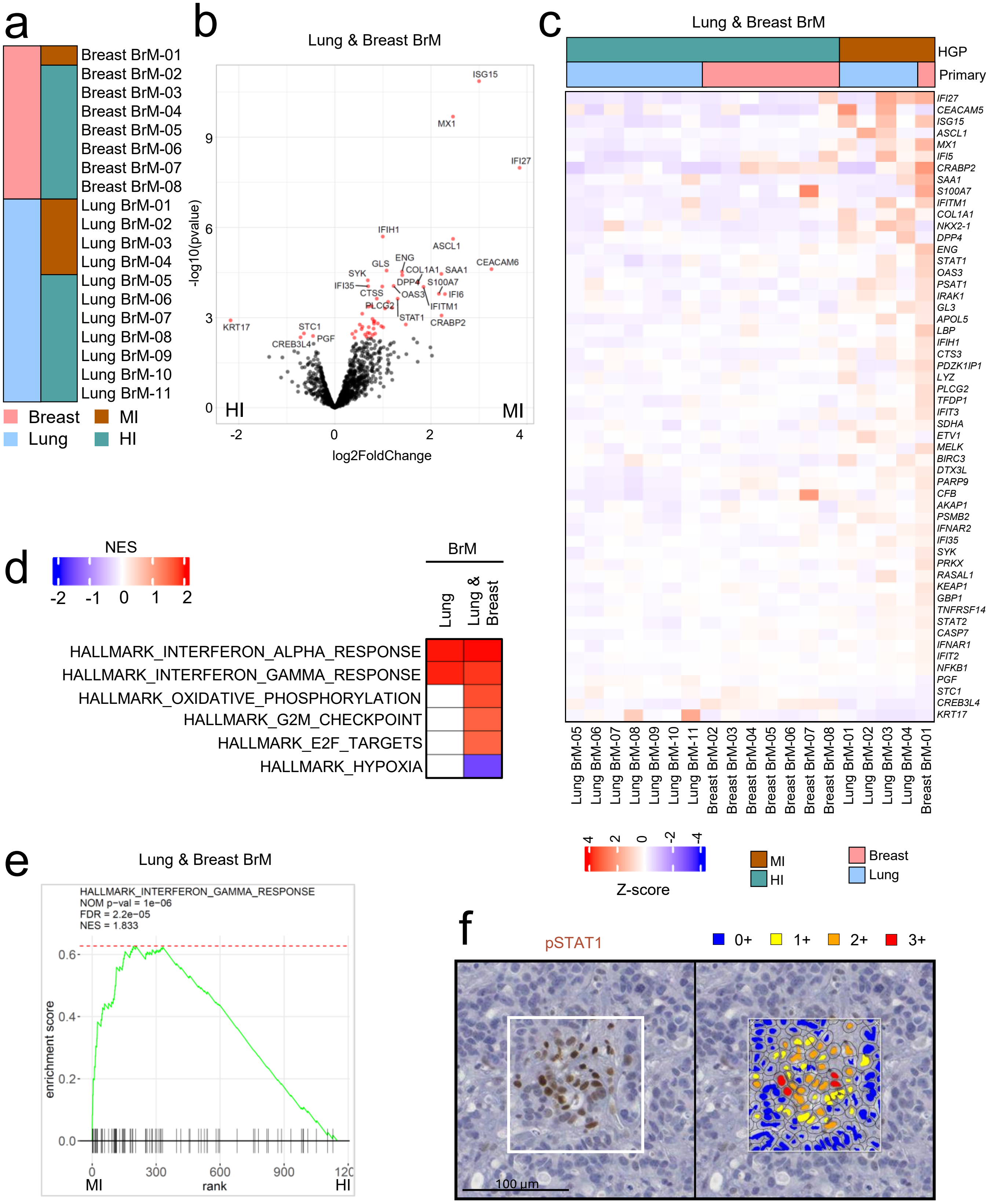

Maritan *et al.* Extended Data Fig. 2

Extended Data Fig. 2. Gene set enrichment analyses of minimally and highly invasive brain metastases. a, Samples used in the Nanostring Digital Spatial Profiling analysis. Colour codes indicating primary type (breast, *pink*; lung, *light blue*) and histopathological growth pattern (highly invasive, HI, *blue*; minimally invasive, MI, *brown*) are shown. **b,** Volcano plot depicting differentially expressed genes (*red*, n = 54) in MI versus highly invasive HI samples in the combined breast and lung cancer BrM cohort. Genes to the left of centre are upregulated in HI samples, while genes to the right of centre are upregulated in MI samples. **c,** Heatmap depicting normalized gene expression (Z-score of log10 normalized gene counts) of differentially expressed genes (padj < 0.1; n = 54) in HI (*blue*) and MI (*brown*) BrM from the combined lung and breast cancer cohort. Primary site is depicted by colour (breast, *pink*; lung, *light blue*). **d,** Heatmap depicting normalized enrichment scores (NES) of gene set enrichment pathway analyses of the lung BrM cohort (*left column*) or combined lung and breast BrM cohort (*right column*). Pathways upregulated in MI samples relative to HI samples are shown in red; pathways downregulated in MI samples relative to HI samples are shown in blue. **e,** Gene set enrichment analysis enrichment plot for the IFN-γ response pathway in the combined lung and breast cancer BrM cohort. **f,** Example of the algorithm used with HALO imaging analysis software to quantify pSTAT1 staining (*brown*) as 0+, 1+, 2+, or 3+. Scale bar: 100 μm. **g,** Quantification of pSTAT1 staining (H-Score) in tumour cells in patient-matched core and margin regions of brain metastases from lung cancer (n = 8 HI, 7 MI), corresponding to Fig. 2f. H-Scores were calculated by multiplying the staining intensity scores (0-3) by the percentage of positively stained tumour cells (1-100%) for a maximum H-Score of 300. For each patient tissue, 10 ROIs (200,000 μm^2 each) were captured at the brain-tumour interface and 10 ROIs were captured within the metastasis core (more than 1.5 mm distance from any brain-tumour interface). Margin:Core H-Score ratio was calculated per sample.

Extended Data Table 1: Differentially expressed genes between HI and MI BrM in the lung cancer cohort

Gene Name	log2FoldChange	pvalue	padj
IFI27	3.876858	9.70E-07	6.29E-04
ISG15	2.895673	1.24E-06	6.29E-04
ASCL1	2.920815	1.37E-05	0.004413
MX1	1.610491	1.74E-05	0.004413
CEACAM6	3.761718	4.18E-05	0.0085
DPP4	1.755709	6.19E-05	0.010486
IFI6	2.64077	7.25E-05	0.010528
ETV4	0.84124	2.52E-04	0.028571
S100A9	-3.76918	2.53E-04	0.028571
COL1A1	1.985331	3.59E-04	0.033711
ETV1	1.109145	3.65E-04	0.033711
PCK2	0.783302	5.34E-04	0.045198
KRT17	-3.02117	5.99E-04	0.046848
IRF7	1.063213	6.81E-04	0.049389
SERPINA1	2.091756	8.81E-04	0.059666
GDF15	1.540682	0.001097	0.069645
KRT5	-3.14179	0.00129	0.077071
FBP1	0.627775	0.001388	0.078356
AREG	1.674199	0.001936	0.099084
IFI35	0.648216	0.00195	0.099084
IFIH1	0.705292	0.002063	0.099806

Extended Data Table 2: Differentially expressed genes between HI and MI BrM in the combined lung and breast cancer cohort

Gene Name	log2FoldChange	pvalue	padj
ISG15	3.002245	1.37E-11	1.58E-08
MX1	2.459778	2.08E-10	1.19E-07
IFI27	3.846451	1.05E-08	4.02E-06
ASCL1	2.461451	2.44E-06	5.60E-04
IFIH1	0.99823	2.04E-06	5.60E-04
CEACAM6	3.261666	2.44E-05	0.004245
ENG	1.398461	2.96E-05	0.004245
GLS	1.077553	2.71E-05	0.004245
DPP4	1.403544	3.85E-05	0.004424
SAA1	2.218098	3.51E-05	0.004424
SYK	0.686756	5.72E-05	0.005974
COL1A1	1.731846	6.82E-05	0.006523
CTSS	0.986682	9.21E-05	0.006808
IFI35	0.693142	9.06E-05	0.006808
IFITM1	1.846059	9.49E-05	0.006808
OAS3	1.223266	8.84E-05	0.006808
IFI6	2.292136	1.64E-04	0.010477
S100A7	2.165966	1.61E-04	0.010477
PLCG2	0.873468	2.33E-04	0.013479
STAT1	1.308241	2.35E-04	0.013479
IRAK1	1.109771	2.98E-04	0.01627
IFNAR2	0.701419	4.29E-04	0.021401
PARP9	0.767066	4.12E-04	0.021401
PSAT1	1.192084	4.70E-04	0.022475
APOL6	1.044335	4.95E-04	0.022717
TNFRSF14	0.570138	7.42E-04	0.032782
CRABP2	2.22146	8.49E-04	0.036116
DTX3L	0.783782	0.00111	0.045494
KRT17	-2.17063	0.001233	0.048792
IFIT3	0.824951	0.001306	0.049971
ETV1	0.811146	0.001369	0.050713
LYZ	0.894689	0.001563	0.055327
SDHA	0.815098	0.00159	0.055327
CASP7	0.519204	0.00171	0.056103
NKX2-1	1.477607	0.001695	0.056103
PDZK1IP1	0.967404	0.00193	0.061549
GBP1	0.573985	0.002022	0.061919
LBP	1.006525	0.002104	0.061919
PSMB2	0.70609	0.002078	0.061919
MELK	0.807237	0.002319	0.066565
STAT2	0.55643	0.002403	0.067274
IFNAR1	0.434754	0.002871	0.078486
AKAP1	0.721859	0.00333	0.084829
NFKB1	0.363811	0.003473	0.084829
PRKX	0.684084	0.003198	0.084829
STC1	-0.64358	0.003356	0.084829
TFDP1	0.838522	0.00341	0.084829
KEAP1	0.629455	0.003652	0.087332
BIRC3	0.790514	0.003754	0.087959
PGF	-0.45453	0.004087	0.093831
RASAL1	0.661416	0.004319	0.097221
CREB3L4	-0.71431	0.004516	0.099705
CFB	0.747041	0.004703	0.099978
IFIT2	0.407551	0.004675	0.099978

Extended Data Table 3: Gene set enrichment analyses of MI and HI BrM

Lung BrM Cohort				
**Pathway**	**pval**	**padj**	**ES**	**NES**
HALLMARK_INTERFERON_ALPHA_RESPONSE	2.68E-05	0.000469	0.718023	1.965227
HALLMARK_INTERFERON_GAMMA_RESPONSE	4.55E-06	0.000159	0.630427	1.933621
HALLMARK_OXIDATIVE_PHOSPHORYLATION	0.033329	0.233304	0.544538	1.490398
HALLMARK_UV_RESPONSE_UP	0.094894	0.416954	0.490129	1.348916
HALLMARK_XENOBIOTIC_METABOLISM	0.109881	0.416954	0.536793	1.347462
HALLMARK_MYC_TARGETS_V1	0.130395	0.416954	0.493244	1.309212
HALLMARK_ALLOGRAFT_REJECTION	0.126885	0.416954	0.42529	1.270277
HALLMARK_G2M_CHECKPOINT	0.14342	0.418307	0.410107	1.248209
HALLMARK_TNFA_SIGNALING_VIA_NFKB	0.176415	0.474965	0.388246	1.206156
HALLMARK_IL6_JAK_STAT3_SIGNALING	0.240206	0.56048	0.419648	1.178757
HALLMARK_E2F_TARGETS	0.235414	0.56048	0.385794	1.164328
HALLMARK_ESTROGEN_RESPONSE_EARLY	0.360313	0.663734	0.404987	1.089511
HALLMARK_APOPTOSIS	0.355121	0.663734	0.357768	1.077071
HALLMARK_ANDROGEN_RESPONSE	0.467474	0.779123	0.429664	1.015728
HALLMARK_UV_RESPONSE_DN	0.456685	0.779123	0.370678	1.014545
HALLMARK_COAGULATION	0.541175	0.811565	0.35549	0.95635
HALLMARK_ESTROGEN_RESPONSE_LATE	0.602877	0.811565	0.323494	0.916755
HALLMARK_NOTCH_SIGNALING	0.598063	0.811565	0.380392	0.91078
HALLMARK_KRAS_SIGNALING_UP	0.647805	0.835056	0.311321	0.889742
HALLMARK_WNT_BETA_CATENIN_SIGNALING	0.677684	0.835056	0.344193	0.855113
HALLMARK_DNA_REPAIR	0.691903	0.835056	0.328617	0.847814
HALLMARK_P53_PATHWAY	0.744739	0.844597	0.288111	0.826197
HALLMARK_ADIPOGENESIS	0.748071	0.844597	0.316894	0.802418
HALLMARK_GLYCOLYSIS	0.819935	0.896804	0.268087	0.771326
HALLMARK_INFLAMMATORY_RESPONSE	0.897665	0.924067	0.240089	0.712965
HALLMARK_MITOTIC_SPINDLE	0.886505	0.924067	0.25983	0.681048
HALLMARK_PI3K_AKT_MTOR_SIGNALING	0.971361	0.971361	0.205713	0.58991
HALLMARK_MTORC1_SIGNALING	0.601725	0.811565	-0.26177	-0.91787
HALLMARK_IL2_STAT5_SIGNALING	0.514365	0.811565	-0.26806	-0.96783
HALLMARK_APICAL_JUNCTION	0.341628	0.663734	-0.30014	-1.06366
HALLMARK_UNFOLDED_PROTEIN_RESPONSE	0.279751	0.611955	-0.41761	-1.15651
HALLMARK_MYOGENESIS	0.131043	0.416954	-0.44426	-1.31973
HALLMARK_HYPOXIA	0.045191	0.263615	-0.37421	-1.39864
HALLMARK_COMPLEMENT	0.03245	0.233304	-0.40899	-1.46363
HALLMARK_EPITHELIAL_MESENCHYMAL_TRANSITION	0.025132	0.233304	-0.39994	-1.48287

Lung and Breast BrM Combined Cohort				
**Pathway**	**pval**	**padj**	**ES**	**NES**
HALLMARK_INTERFERON_ALPHA_RESPONSE	4.80E-07	1.25E-05	0.719883	1.990726
HALLMARK_INTERFERON_GAMMA_RESPONSE	6.77E-07	1.25E-05	0.627532	1.8433
HALLMARK_OXIDATIVE_PHOSPHORYLATION	0.001355	0.016715	0.629864	1.692706
HALLMARK_G2M_CHECKPOINT	0.00204	0.018866	0.531159	1.547121
HALLMARK_E2F_TARGETS	0.004514	0.031425	0.530903	1.529242
HALLMARK_MYC_TARGETS_V1	0.057642	0.236971	0.536487	1.405106
HALLMARK_UV_RESPONSE_UP	0.053097	0.236971	0.510891	1.38757
HALLMARK_COAGULATION	0.103319	0.347528	0.488813	1.317974
HALLMARK_ALLOGRAFT_REJECTION	0.099327	0.347528	0.436111	1.270272
HALLMARK_ESTROGEN_RESPONSE_LATE	0.165723	0.49647	0.439404	1.228477
HALLMARK_MITOTIC_SPINDLE	0.211647	0.559352	0.466597	1.215917
HALLMARK_COMPLEMENT	0.174435	0.49647	0.427053	1.213308
HALLMARK_XENOBIOTIC_METABOLISM	0.302253	0.602912	0.445361	1.145119
HALLMARK_IL6_JAK_STAT3_SIGNALING	0.286858	0.602912	0.412869	1.141727
HALLMARK_ADIPOGENESIS	0.309603	0.602912	0.444989	1.137008
HALLMARK_TNFA_SIGNALING_VIA_NFKB	0.309546	0.602912	0.373974	1.100839
HALLMARK_ESTROGEN_RESPONSE_EARLY	0.357816	0.630437	0.406634	1.096397
HALLMARK_P53_PATHWAY	0.390946	0.6575	0.380583	1.069444
HALLMARK_APOPTOSIS	0.423427	0.675459	0.360342	1.045037
HALLMARK_KRAS_SIGNALING_UP	0.438136	0.675459	0.368396	1.041331
HALLMARK_DNA_REPAIR	0.491087	0.726809	0.392352	1.002515
HALLMARK_ANDROGEN_RESPONSE	0.545935	0.751496	0.409182	0.963138
HALLMARK_INFLAMMATORY_RESPONSE	0.59845	0.751496	0.33218	0.946444
HALLMARK_KRAS_SIGNALING_DN	0.604834	0.751496	0.390144	0.918327
HALLMARK_MTORC1_SIGNALING	0.63522	0.751496	0.328288	0.917822
HALLMARK_UV_RESPONSE_DN	0.632749	0.751496	0.338347	0.915512
HALLMARK_PI3K_AKT_MTOR_SIGNALING	0.661404	0.751496	0.319932	0.902525
HALLMARK_UNFOLDED_PROTEIN_RESPONSE	0.645078	0.751496	0.375908	0.884819
HALLMARK_NOTCH_SIGNALING	0.670253	0.751496	0.360099	0.867292
HALLMARK_WNT_BETA_CATENIN_SIGNALING	0.724115	0.788007	0.332488	0.827847
HALLMARK_TGF_BETA_SIGNALING	0.7784	0.82288	0.324994	0.774428
HALLMARK_MYOGENESIS	0.812262	0.834825	0.296319	0.751061
HALLMARK_GLYCOLYSIS	0.910227	0.910227	0.243924	0.679419
HALLMARK_IL2_STAT5_SIGNALING	0.351648	0.630437	-0.23542	-1.07023
HALLMARK_APICAL_JUNCTION	0.292208	0.602912	-0.24538	-1.09561
HALLMARK_EPITHELIAL_MESENCHYMAL_TRANSITION	0.048851	0.236971	-0.2894	-1.33195
HALLMARK_HYPOXIA	0.005096	0.031425	-0.33848	-1.59391

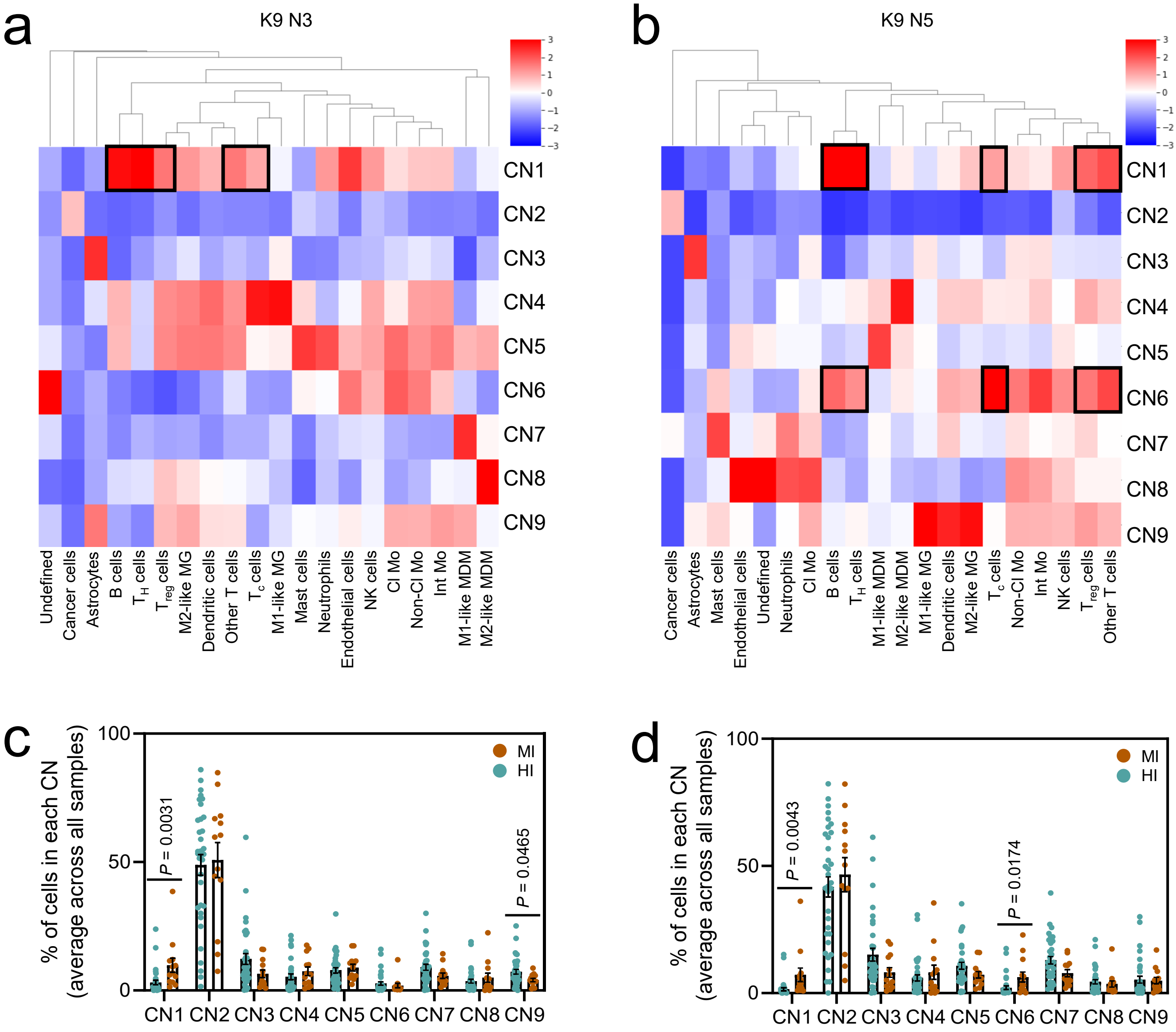

Maritan *et al.* Extended Data Fig. 3

e

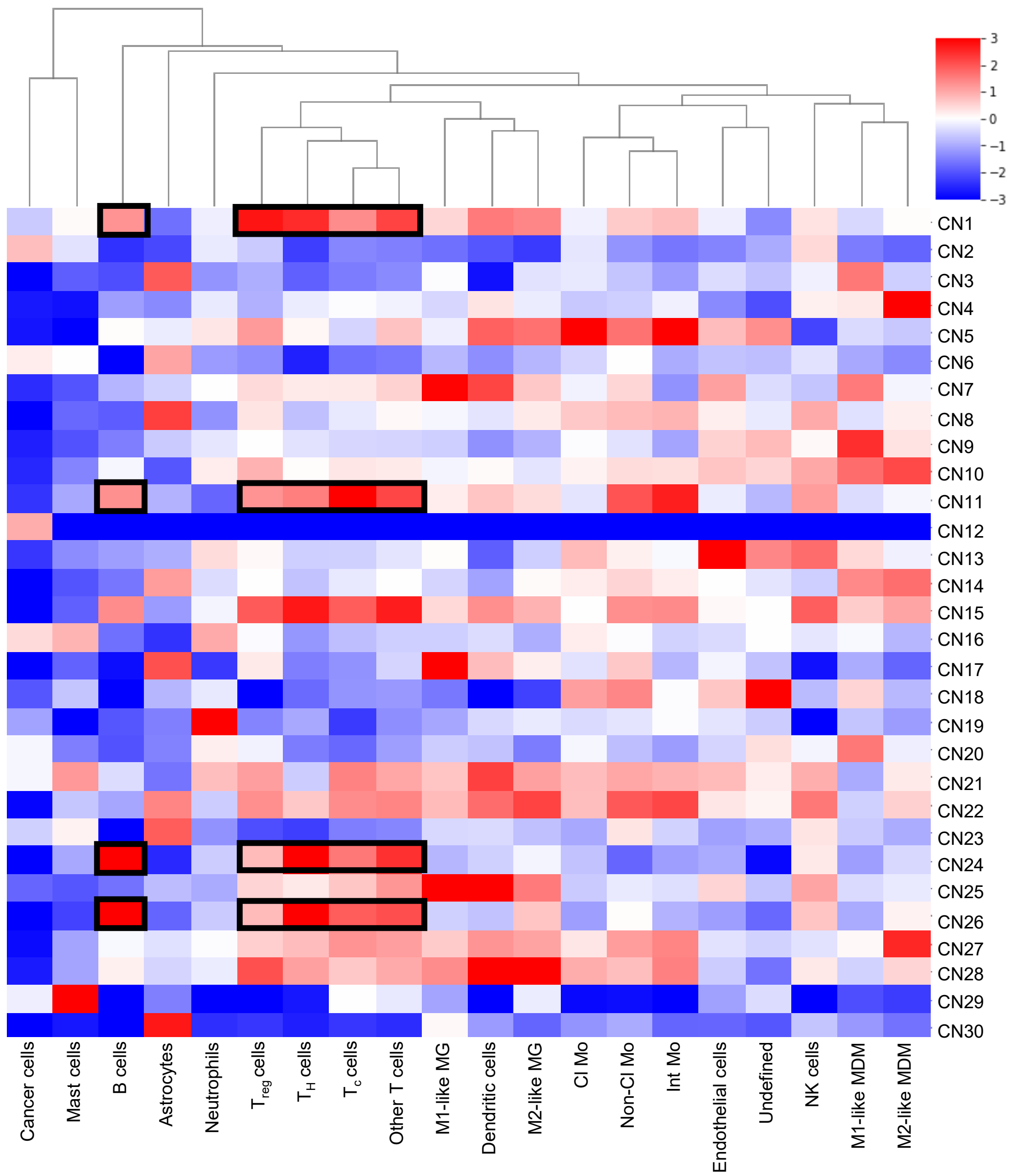

f

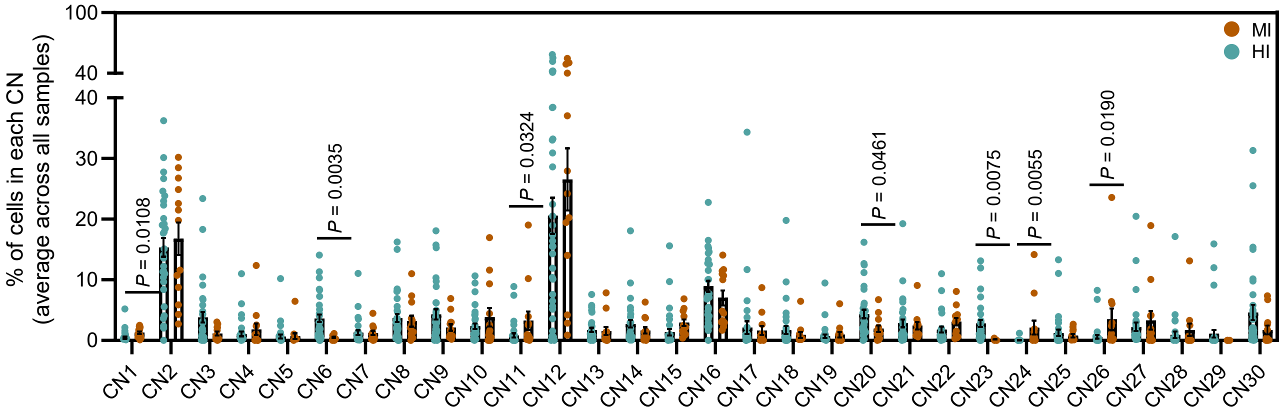

Maritan *et al.* Extended Data Fig. 3, Continued

Extended Data Fig. 3. Cellular neighbourhood analyses of brain metastasis margins with modified neighbourhood parameters. a, b, Heatmap of cell type distribution across 9 cellular neighbourhoods (CN) in BrM margins (n = 47 images) using either $N = 3$ (**a**) or $N = 5$ (**b**) nearest neighbours. Boxes highlight cell types of interest. **c, d,** Bar graph depicting percentage of cells in each CN across HI (*blue*, n = 34 images) and MI (*brown*, n = 13 images) BrM margins, for CNs calculated with $N = 3$ (**c**) or $N = 5$ (**d**) nearest neighbours. P values were calculated using Student's t test. **e,** Heatmap of cell type distribution across 30 CNs in BrM margins (n = 47 images) using $N = 10$ nearest neighbours. Boxes highlight cell types of interest. **f,** Bar graph depicting percentage of cells in each CN (K30, N10) across HI (*blue*, n = 34 images) and MI (*brown*, n = 13 images) BrM margins. P values were calculated using Student's t test.

Extended Data Table 4: Significant pairwise interaction and avoidance behaviours between cell types in MI (brown) and HI (blue) BrM

Interaction	P value	Interaction Score (MI)	Interaction Score (HI)
Cancer - Cl Mo	0.0406	31758.7692	16908.2647
B cells - B cells	0.0003	34333.6154	8576.5588
B cells - T_c cells	0.0022	32331.5385	10522.6176
B cells - T_H cells	0.0001	33772.5385	7858.1765
B cells - M1-like MDM	0.0001	27305.6923	4266.3529
B cells - M2-like MDM	0.0051	28212.6923	9736.7941
Endothelial cells - Other T cells	0.0151	26879.0769	10232.4706
Astrocytes - T_c cells	0.0053	35778.3077	17120.3235
T_c cells - B cells	0.0079	28722.9231	10382.4412
T_c - Endothelial cells	0.0196	27378.2308	11232.6176
T_c cells - T_c cells	0.0217	44885.4615	29396.6176
T_c cells - T_H cells	0.0042	42473.7692	21658.0882
T_c cells - Other T cells	0.0220	34991.3846	17937.4412
T_c cells - M1-like MDM	0.0196	41140.9231	24827.5000
T_c cells – M1-like MG	0.0025	33086.8462	11856.0588
T_c cells - M2-like MG	0.0191	29380.0769	12892.9118
T_H cells - Cancer	0.0343	27333.9231	11745.8824
T_H cells - B cells	0.0001	33663.0000	7551.0294
T_H cells - Endothelial cells	0.0111	31652.6923	14270.8235
T_H cells - Astrocytes	0.0283	29393.7692	13522.5294
T_H cells - T_c cells	0.0009	47440.9231	23758.9412
T_H cells - T_H cells	0.0015	49544.8462	27258.5588
T_H cells - T_{reg} cells	0.0004	27448.9231	5624.0588
T_H cells - Other T cells	0.0000	45676.5385	13374.5294
T_H cells - M1-like MDM	0.0000	49259.3077	20103.4118
T_H cells - M2-like MDM	0.0004	48158.4615	24540.5000
T_H cells - Cl Mo	0.0002	26971.0769	4907.6471
Other T cells - Endothelial cells	0.0001	26474.3077	4780.0882
Other T cells - T_H cells	0.0007	37462.4615	13235.4118
Other T cells - M2-like MDM	0.0212	26656.2308	11447.8529
M1-like MDM - T_H cells	0.0305	40202.4615	25309.6176
M1-like MDM - Other T cells	0.0493	27713.7692	15482.6765
M1-like MDM - Cl Mo	0.0101	35135.0769	18967.4118
M2-like MDM - Cancer	0.0267	12728.1538	28785.8529
M2-like MDM - B cells	0.0186	27294.7692	11652.2353
M2-like MG - Cancer	0.0000	25383.9231	965.7647
M2-like MG - Astrocytes	0.0111	28189.0000	10880.4412
**Avoidance**	**P value**	**Avoidance Score (MI)**	**Avoidance Score (HI)**
M1-like MG - Cancer	0.013702	30471.2308	14814.2059

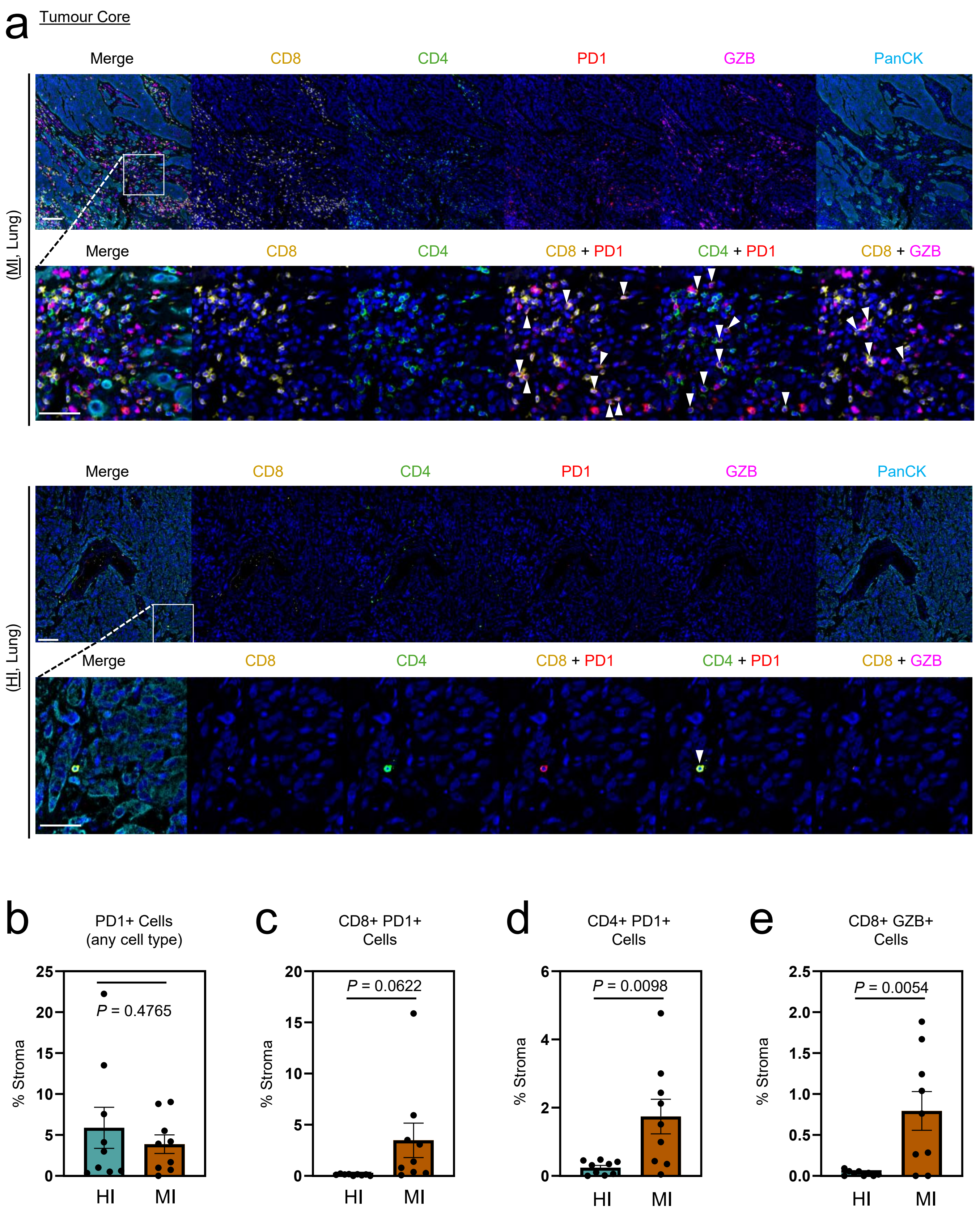

Maritan *et al.* Extended Data Fig. 4

Extended Data Fig. 4. Multiplex immunohistofluorescence (IHF) staining of lung cancer brain metastasis cores. **a**, Representative IHF staining for CD8 (yellow), CD4 (green), PD1 (red), granzyme B (GZB, pink) and pan cytokeratin (PanCK, cyan) in tumour cores of minimally invasive (MI, *top*) and highly invasive (HI, *bottom*) lung cancer brain metastasis patient samples. Scale bars: 100 µm (*low magnification*), 50 µm (*high magnification*). **b-e**, Quantification of $PD1^+$ (**b**), $CD8^+$ $PD1^+$ (**c**), $CD4^+$ $PD1^+$ (**d**), or $CD8^+$ GZB^+ (**e**) cells as a percentage of stroma ($PanCK^-$ cells) in HI and MI BrM samples. P-values were calculated using Student's *t*-test.

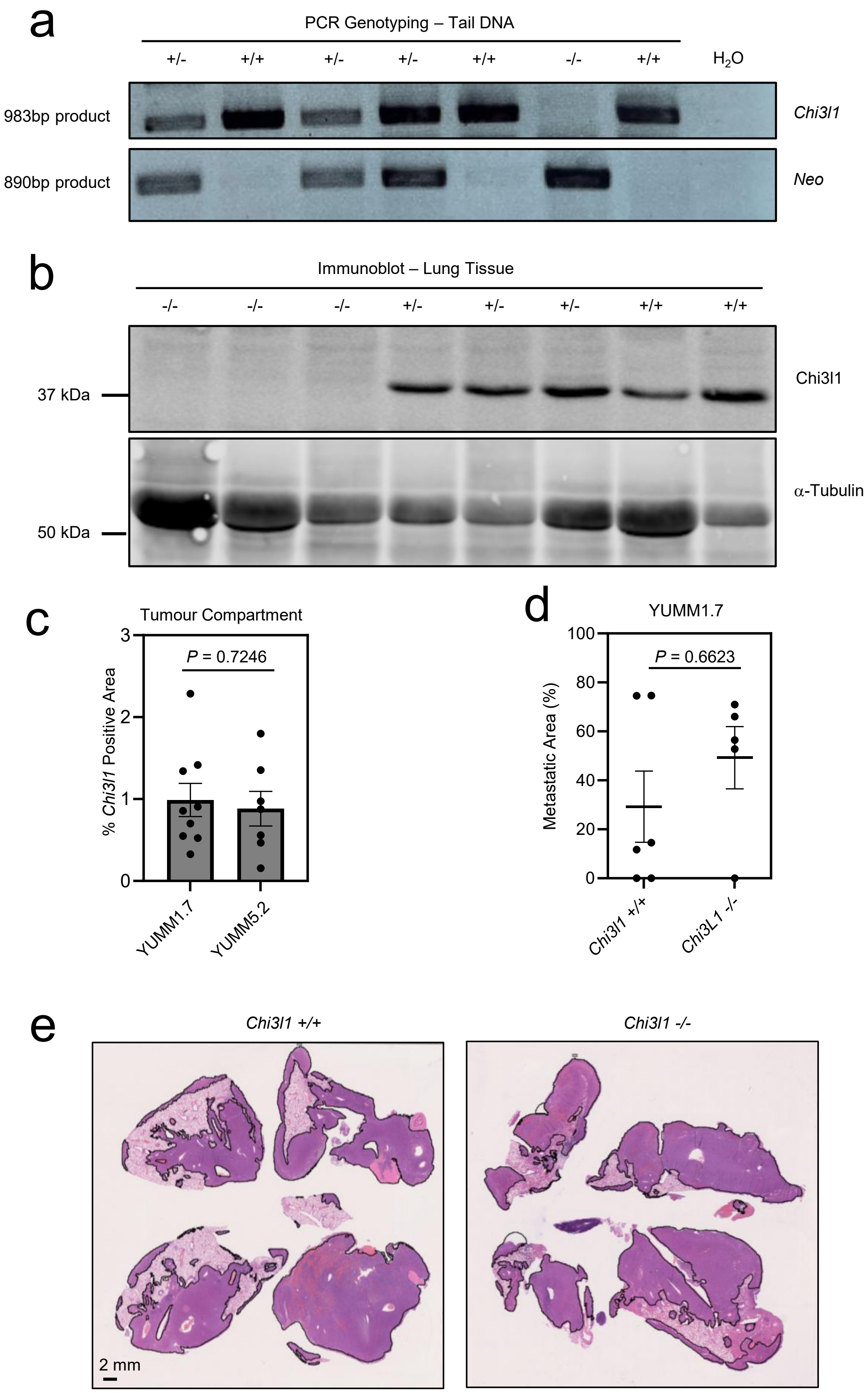

Maritan *et al.* Extended Data Fig. 5

Extended data Fig. 5. Characterization of transgenic mice lacking Chi3l1. a, PCR genotyping of tail DNA from Chi3L1 +/+, +/- and -/- mice. Primers were used to amplify *Chi3L1* or the neomycin cassette. Distilled water (H_2O) was included as a negative control for primer contamination. **b**, Immunoblot analysis of lung tissue confirming the lack of Chi3l1 expression in homozygous mutants (-/-) compared to heterozygous mutants (+/-) or wildtype mice (+/+). a-Tubulin was used as a loading control. **c,** Quantification of RNAscope staining for *Chi3l1* in the tumour compartment of wildtype mice bearing intracranially injected YUMM1.7 or YUMM5.2 syngeneic melanoma cells. P-value was calculated using Student's *t*-test. **d,** Quantification of lung metastatic burden of *Chi3l1* +/+ and -/- mice injected with YUMM1.7 cells into the lateral tail vein. P-value was calculated using a Mann-Whitney test. **e,** Representative H&E images of lungs corresponding to data in **(d)** are shown, with tumour burden annotated by solid black outlines.

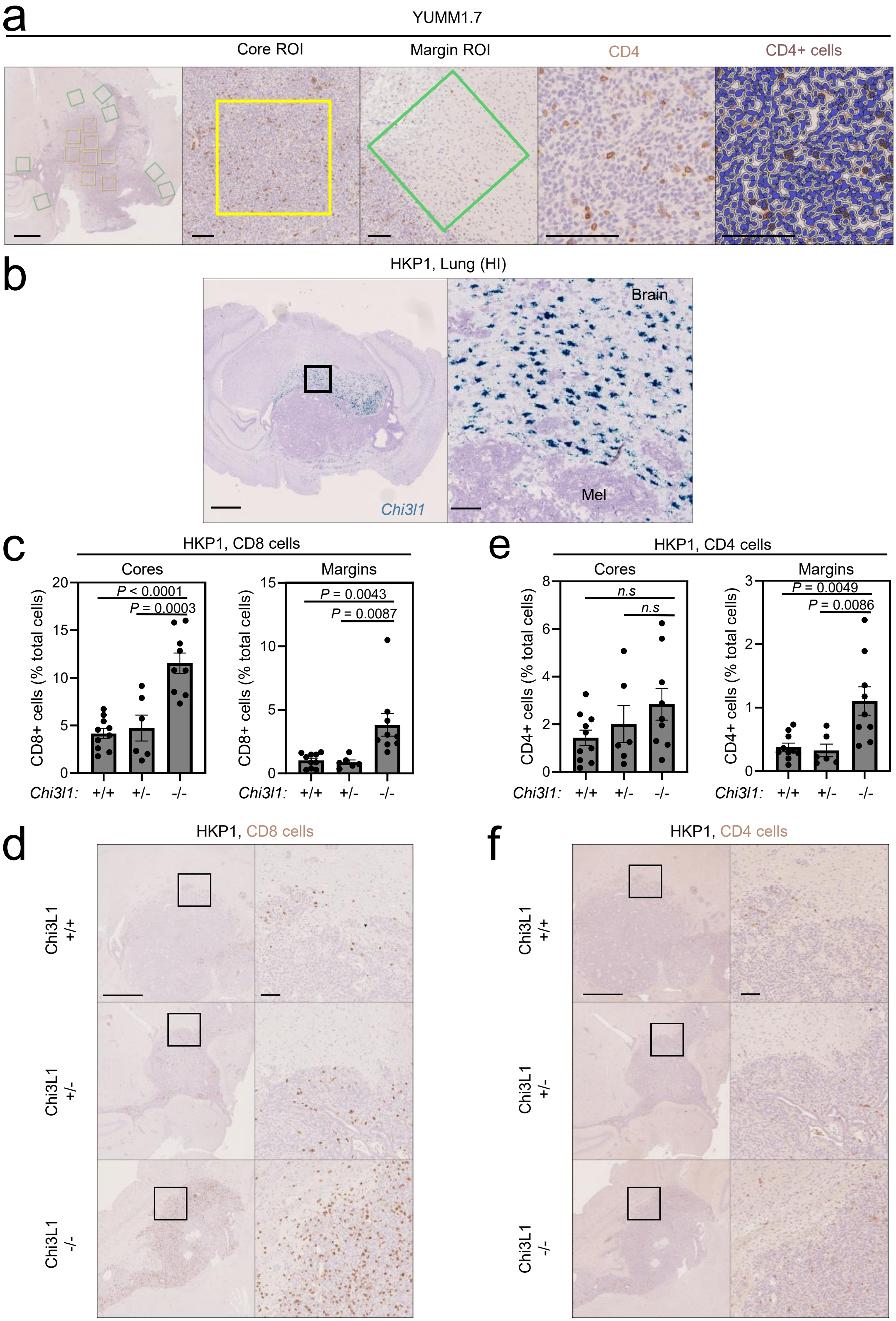

Maritan *et al.* Extended Data Fig. 6

g

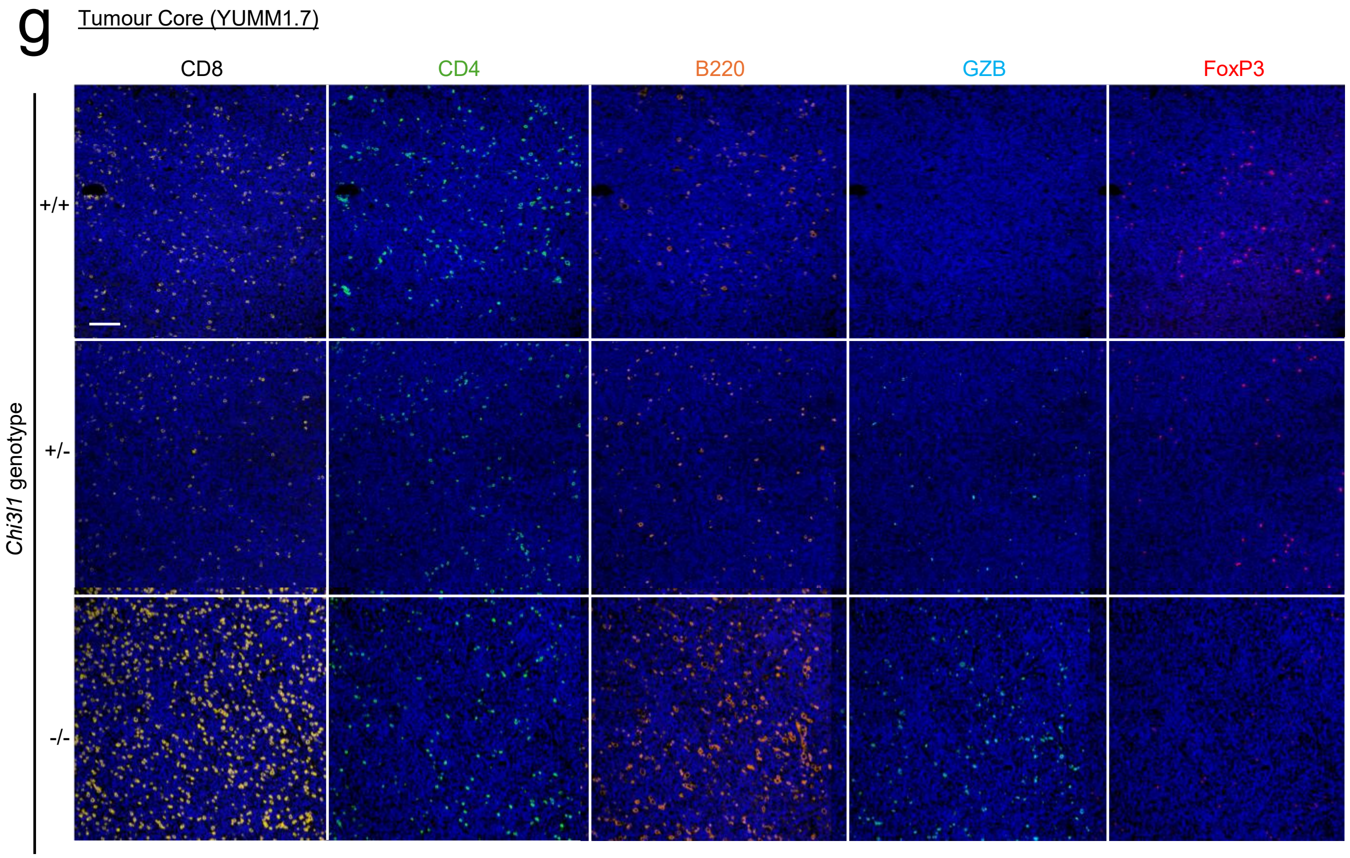

h

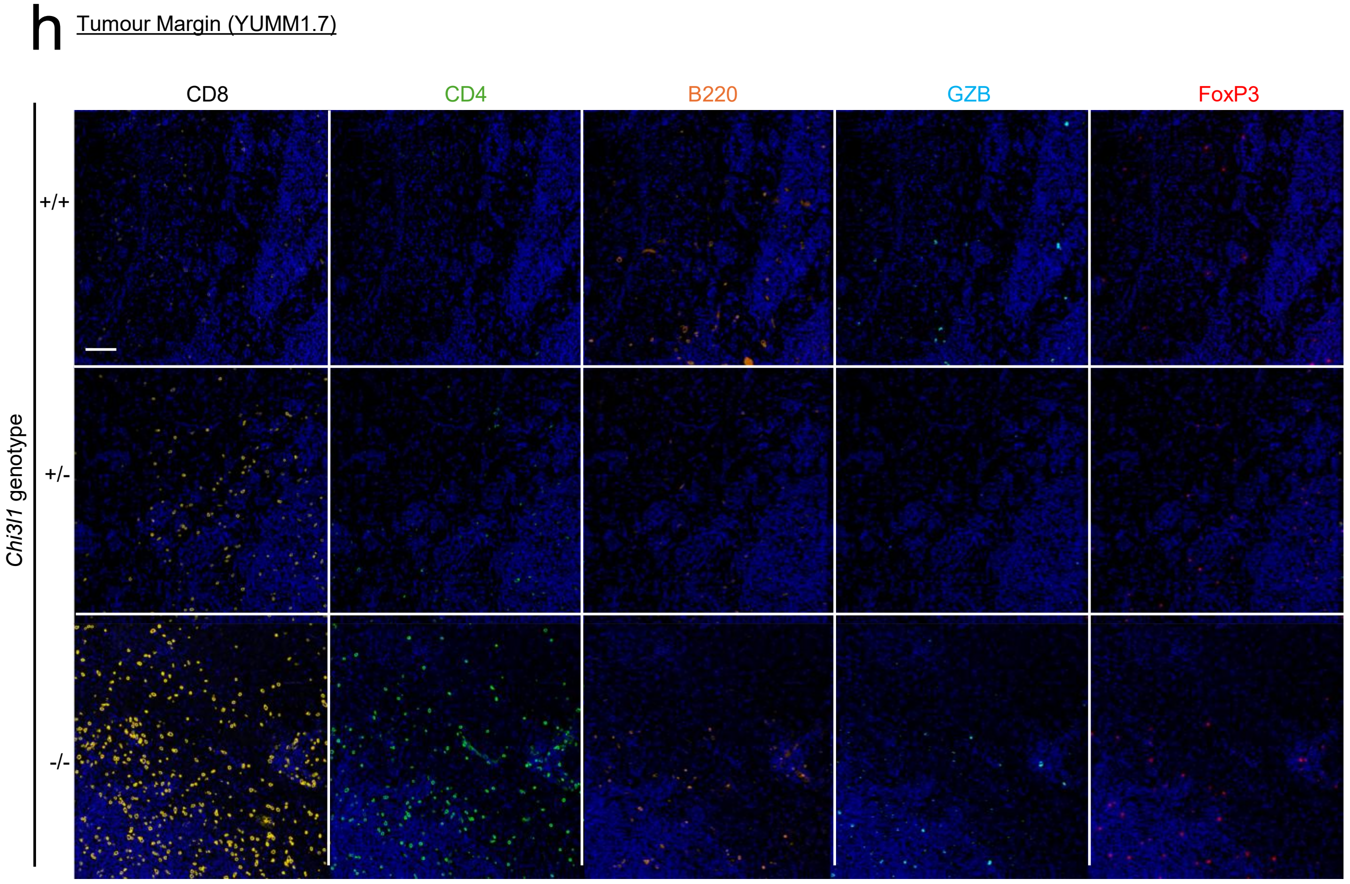

Maritan *et al.* Extended Data Fig. 6, Continued

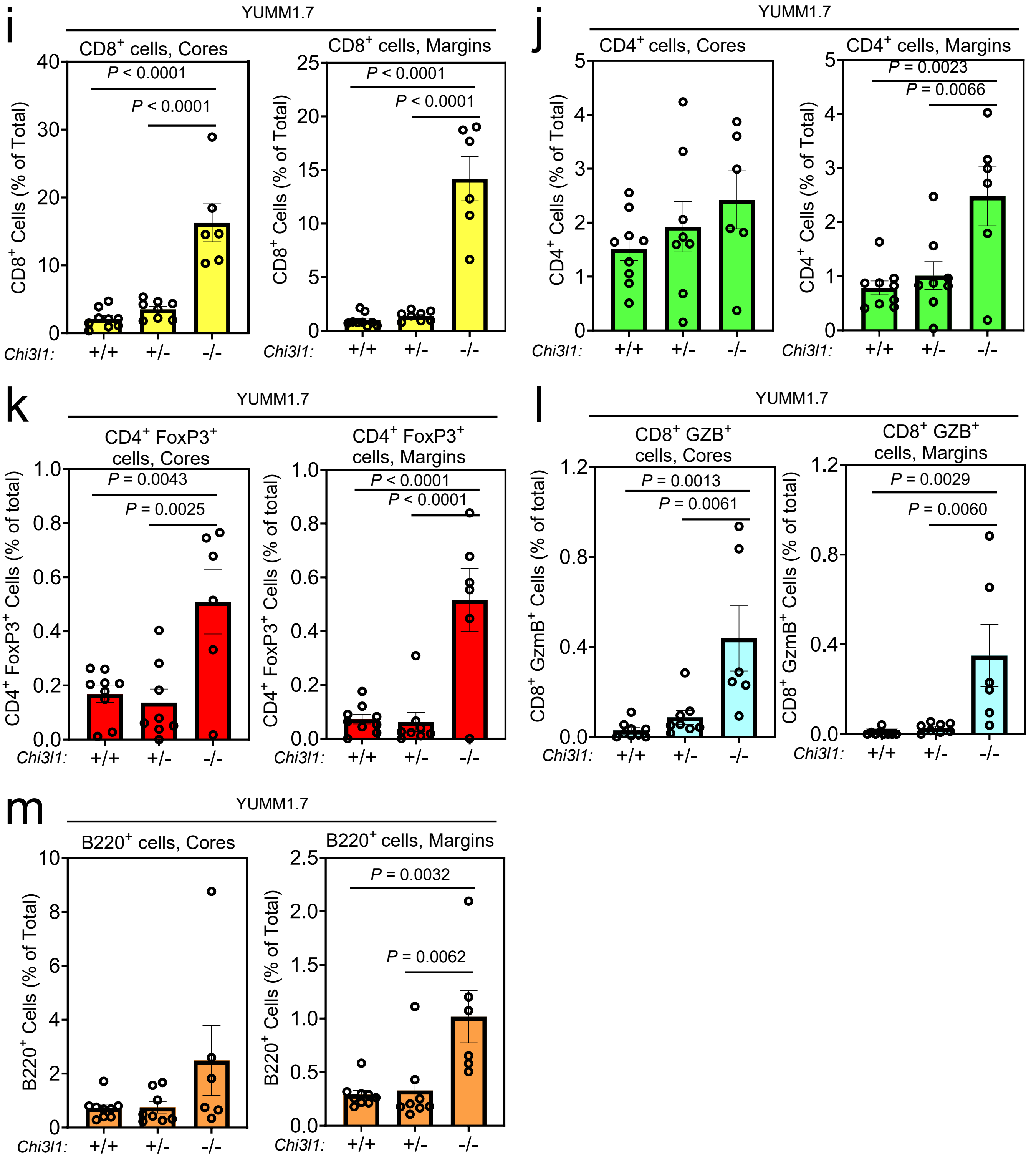

Maritan *et al.* Extended Data Fig. 6, Continued

Extended Data Fig. 6. IHC quantification methodology and immune characterization of highly invasive syngeneic brain metastasis models. **a**, Representative annotated regions (cores, yellow; margins, green) for analysis of CD8 and CD4 immunohistochemical staining corresponding to Fig. 6a-d. CD4 staining is shown as an example of the staining-detection algorithm (*far right*). Scale bars: 1mm (*left*), 100 µm (*others).* **b**, Representative RNAscope staining for *Chi3l1* (blue) in wildtype (WT) mouse brains bearing intracranially injected HKP1 cells (lung, HI). Scale bars: 1 mm (*left*), 100 µm (*right*). **c,** Quantification of $CD8^{+}$ cells in the tumour cores (*left*) and margins (*right*) of *Chi3l1* +/+, +/- and -/- mice bearing intracranially injected HKP1 lung cancer cells. **d,** Representative images of immunohistochemical (IHC) staining for CD8 in HKP1-derived brain lesions. Scale bars: 1 mm (*upper left image*), 100 µm (*upper right image*). **e,** Quantification of $CD4^{+}$ cells in the tumour cores (*left*) and margins (*right*) of *Chi3l1* +/+, +/- and -/- mice bearing intracranially injected HKP1 lung cancer cells. **f,** Representative images of immunohistochemical (IHC) staining for CD4 in HKP1-derived brain lesions. Scale bars: 1 mm (*upper left image*), 100 µm (*upper right image*). **g, h,** Representative images of multiplex IHF staining for CD8 (yellow), CD4 (green), B220 (orange), granzyme B (GZB, blue) and FoxP3 (red) in brains of wildtype (+/+, *top*), heterozygous (+/-, *middle*) or homozygous *Chi3l1* knockout (-/-, *bottom*) mice bearing intracranially injected YUMM1.7 melanoma cells. Images were of tumour cores (**g**) or the tumour-brain margins (**h**). Scale bars: 100 µm. **i-m,** Quantification of frequency (as a percentage of total cells) of CD8+ (**i**), CD4+ (**j**), CD4+ FoxP3+ (**k**), CD8+ GZB+ (**l**), B220+ (**m**) cells, corresponding to images in panels C and D. P values were calculated by one way ANOVA tests. Non-significant values are not shown.
