# Supplementary material for: Distinct immune landscapes characterize highly versus minimally invasive brain metastases
Source: JCI Insight. 2026 May 22;11(10):e199498. doi: 10.1172/jci.insight.199498 (PMC13232719; doi:10.1172/jci.insight.199498)
Supplement: Unedited blot and gel images [file jciinsight-11-199498-s138.pdf]

Extended Data Figure 5A: full unedited gel

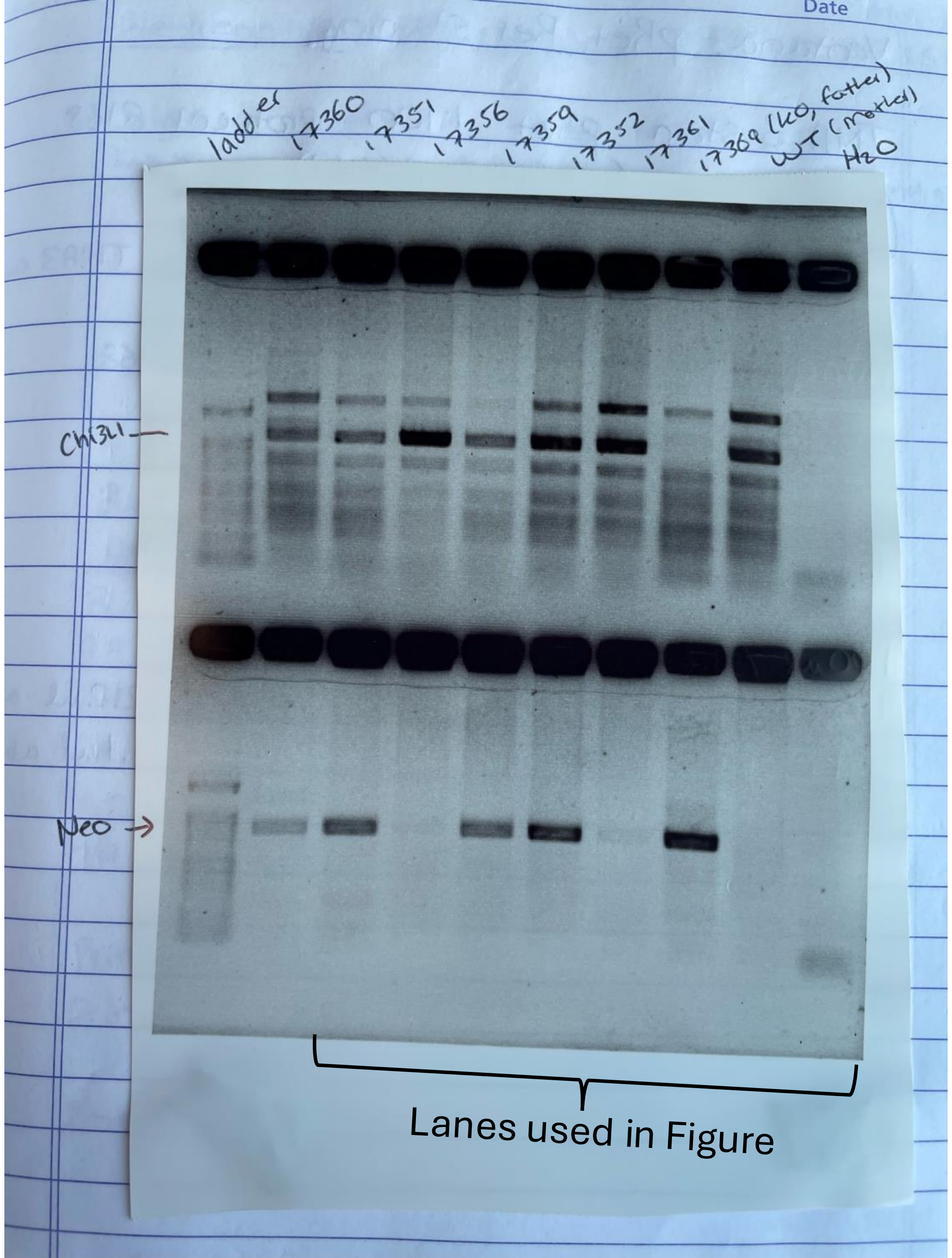

Extended Data Figure 5B: CHI3L1; full unedited gel

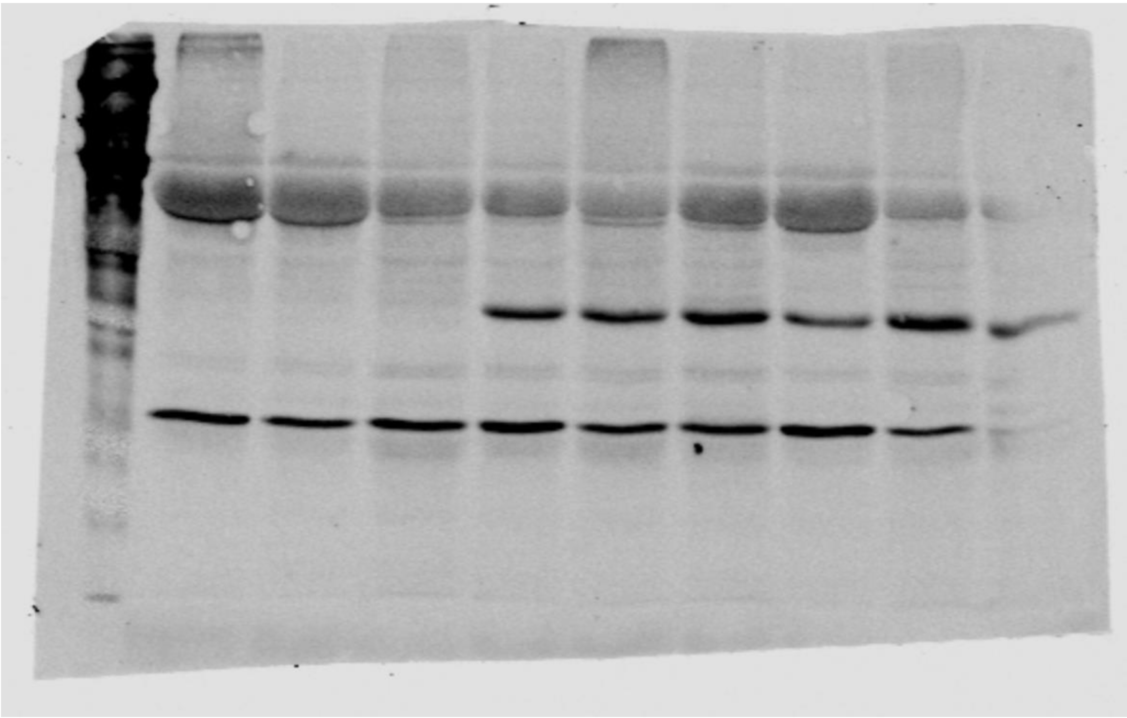

Immunoblot of lung tissue; antibody: Invitrogen, #81355, 1:500

Extended Data Figure 5B: α-Tubulin; full unedited gel

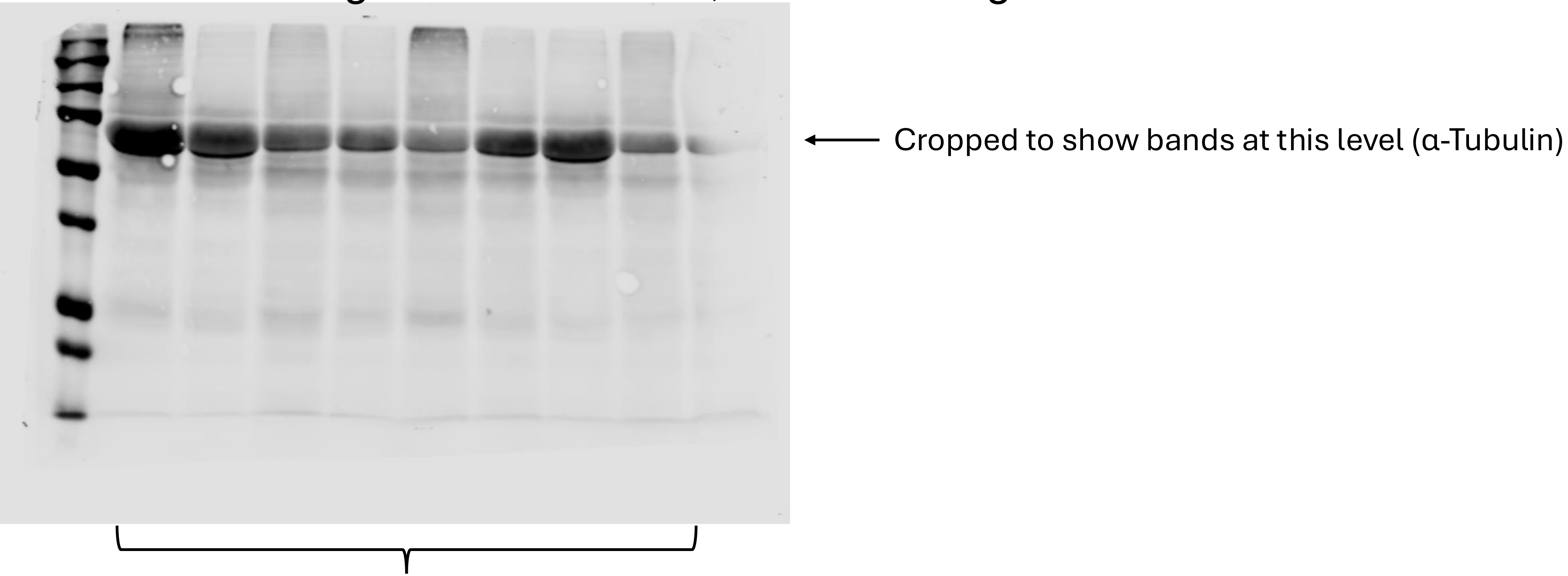

Immunoblot of lung tissue; antibody: Sigma, #T9026, 1:10,000
